# Effectiveness of home-based exercise for functional rehabilitation in older adults after hip fracture surgery: A systematic review and meta-analysis of randomized controlled trials

**DOI:** 10.1371/journal.pone.0315707

**Published:** 2024-12-19

**Authors:** Lijun Zhao, Xiaona Zhao, Bin Dong, Xiaobin Li

**Affiliations:** Department of Orthopaedic Trauma, Yuncheng Central Hospital affiliated to Shanxi Medical University, Yuncheng, Shanxi Province, China; Lorestan University, ISLAMIC REPUBLIC OF IRAN

## Abstract

This systematic review and meta-analysis was performed to assess effectiveness of home-based exercise compared with control interventions for functional rehabilitation in elderly patients after hip fracture surgery. Comprehensive literature search was performed on PubMed, EMBASE, Web of Science, Cochrane library, and Clinicaltrails.gov to identify eligible randomized controlled trials (RCTs). Standard mean difference (SMD) and risk ratio (RR) with 95% confidence interval (CI) was calculated. The certainty of evidence of each outcome was assessed by using Grading of Recommendations Assessment, Development and Evaluation (GRADE) approach. A total of 28 articles reporting 21 unique RCTs (n = 2470) were finally included. Compared with control interventions, home-based exercise significantly improved Berg balance scale (BBS, SMD = 0.28, 95%CI: 0.03 to 0.53, P = 0.030), timed-up-and-go test (TUG, SMD = -0.28, 95%CI: -0.50 to -0.07, P = 0.009), Short Fort-36 physical component score (SF-36 PCS, SMD = 0.49, 95%CI: 0.28 to 0.70, P<0.001), and knee extensor strength (SMD = 0.23, 95%CI: 0.09 to 0.37, P = 0.001). No significant improvement was observed in gait speed, 6-minute walking test, short physical performance battery performance (SPPB), activities of daily living (ADL), or fear of falling in the home exercise group. Risk of adverse events, including emergency department visits, hospital readmissions, and falls, did not differ between both groups. According to GRADE, the overall certainty of evidence was moderate for usual gait speed, SPPB, ADL, fear of falling, and SF-36 PCS, and was low or very low for the other outcomes. Our meta-analysis demonstrated home-based exercise had positive effect on physical function after hip fracture surgery. Home-based rehabilitation might be recommended for rehabilitation of fractured patients after hospital discharge.

## Introduction

Hip fracture is a major traumatic injury and a devastating disease for older adults, and the incidence increases with age in older population aged 65 or above [1–3]. The number of hip fractures each year is expected to increase to 4.5 million by 2050 [4]. Hip fracture limits the independence of activities of daily living (ADL) and significantly reduces physical activity and quality of life during inpatient period and for a long time after discharge [5]. It is associated with high disability and mortality in older adults with a 1-year mortality rate of approximately 14–30% [6]. Despite surgical treatment, only 40–60% of fractured patients restore their pre-fracture level of mobility, and 40–70% recover level of ADL’S independence [7]. The impact of hip fractures on physical function and quality of life is long-lasting, and many patients still need long-term nursing care in an institution or at home after hip fractures [8].

Structured post-surgery rehabilitation is critical for the recovery of physical function and self-care ability. Recent meta-analyses demonstrated that postoperative exercise significantly improved mobility, ADL, muscle strength and balance in older adults after fracture surgery [9, 10]. However, due to the shortage of beds and high cost for supervised institution-based exercise, most of fractured patients will eventually return home. Thus, apart from acute or subacute in-hospital rehabilitation, post-discharge exercise is still warranted for long-term functional recovery. More and more attention is given to home-based rehabilitation programs to extend rehabilitation guidance from the hospital to the home. Home-based exercise with high feasibility, low cost, and incorporation with daily-life function, is recommended as an alternative for rehabilitation and is suitable for frail patients who cannot attend training programs outside the home [11]. Yet, the effectiveness of home-based exercise in older adults after hip fracture surgery is still in controversy. Several randomized controlled trials (RCTs) showed superior performance of home-based exercise than control interventions by reducing hospital stay and fear of falling, and improving physical activity, mobility, and independence of ADL [12–16]. The other trials failed to find significant differences of functional recovery between home-based rehabilitation and control interventions such as standard care or active controls [17, 18].

Considering the conflicting results of published RCTs, we performed the present systematic review and meta-analysis to investigate the effectiveness of home-based exercise on balance, mobility, independence in ADL, lower extremities strength, quality of life, and fear of falling in older adults after hip fracture surgery.

## Methods

### Literature search and selection

This meta-analysis was conducted in compliance with the Preferred Reporting Items for Systematic review and meta-analysis guideline (S1 Checklist) [19]. Electronic literature databases, including PubMed, EMBASE, Web of Science, Cochrane library, and Clinicaltrials.gov, were comprehensively searched from inception to June 31st, 2023 to identify potentially relevant articles. The following search terms were used: (hip fracture* OR femoral fracture*) AND (home-based OR in-home OR home) AND (rehabilitation OR exercise OR physiotherapy OR training). The reference lists of relevant reviews, meta-analyses and research articles were manually reviewed to identify additional eligible articles. There was no language restriction.

Eligible studies were selected according to PICOS framework as follows. Participant (P): older adults after hip fracture surgery. Intervention (I): home-based rehabilitation. Control (C): other exercise, rehabilitation or usual care. Outcome (O): mobility, balance, activities of daily living (ADL), muscle strength, quality of life, and adverse events including emergency department visits, falls, and hospital readmissions. Study design (S): RCTs. Reviews, case reports, studies with incomplete or missing data or inappropriate controls, and non-randomized studies were all excluded.

### Outcome measurements

Balance was measured by Berg balance score (BBS) and timed-up-and-go test (TUG). Independence in ADL was assessed using Barthel index and instrumental ADL. Mobility indexes included fast and usual gait speed, 6-minute walking test (6MWT), short physical performance battery (SPPB), and walking outdoors. Fear of falling was assessed by falls efficacy scale. Knee extensor strength was measured for muscle strength. Quality of life was assessed using Short Form-36 (SF-36) questionnaire physical component score (PCS) and mental component score (MCS). Adverse events included emergency department visits, falls, and hospital readmissions.

### Risk of bias assessment

The Cochrane Collaboration’s tool for assessing risk of bias (RoB) was used to assess the risk of bias in terms of random sequence generation, allocation concealment, blinding of participants and personnel, blinding of outcome assessment, incomplete outcome data, selective reporting, and other bias. The risk of bias of each domain was judged as low, unclear or high.

### Data extraction

The following information of each study included in meta-analysis was extracted: first author, publication year, country, sample size, mean age, percentage of females, activities and component of home-based rehabilitation and control, initiation time and duration of interventions, time points of outcome assessment, and outcome measurements. Two independent authors performed literature search and selection, risk of bias assessment, and data extraction. Discrepancies, if occurring, were resolved by further discussion.

### Statistical analysis

Statistical analysis was conducted by using STATA 16.0 (Stata Corporation, TX, USA). The heterogeneity was assessed by using I^2^ statistic and Q test. I^2^ < 50% and P value of Q test > 0.10 indicated low between-study heterogeneity, and a fixed-effect model was used for quantitative synthesis. Otherwise, a random-effect model was used for meta-analysis with high heterogeneity. Standard mean difference (SMD), measured by cohen’s d, with 95% confidence interval (CI) were calculated for pooled effect size of continuous variables. The effect size of SMD was categorized as small (0.1 to 0.4), medium (0.5–0.7) or large (0.8 or greater) [20]. Risk ratio (RR) with 95%CI was calculated to assess the association strength between home-based exercise and categorical variable outcomes including walking outdoors, emergency department visits, falls, and hospital readmissions. We noted that several trials measured outcomes repeatedly at different time point during follow-up. Thus, the outcome measurements of the longest follow-up were synthesized for overall analysis. Besides, outcomes measured at a time point of ≤ 6 months of follow-up were pooled for short-term effect analysis, whereas those measured at a time point of > 6 months of follow-up were synthesized for long-term effect analysis. Further subgroup analysis were performed according to intervention initiation time after surgery (≤3 months, >3 months), intervention duration (≤3 months, >3 months), and rehabilitation type (multicomponent, exercise only). Here, “multicomponent home-based rehabilitation” was defined as programs including components of exercise, education, and evaluation and modification of environment while “exercise only” was defined as those including only home exercise [21]. Sensitivity analysis applying a Leave-One-Out method was performed to evaluate the robustness of pooled results [22–24]. Potential publication bias was assessed by funnel plot and Egger’s test. P value < 0.05 was considered statistically significant.

### Certainty of evidence

Two independent authors assessed the certainty of evidence of pooled results using the Grading of Recommendations Assessment, Development and Evaluation (GRADE) approach [25]. The overall certainty of evidence was graded as very low, low, moderate, and high by evaluating the certainty in terms of study design, risk of bias, inconsistency, indirectness, imprecision and publication bias. Conflicts were resolved by further discussion.

## Results

### Baseline characteristics of trials included in meta-analysis

**Fig 1:**
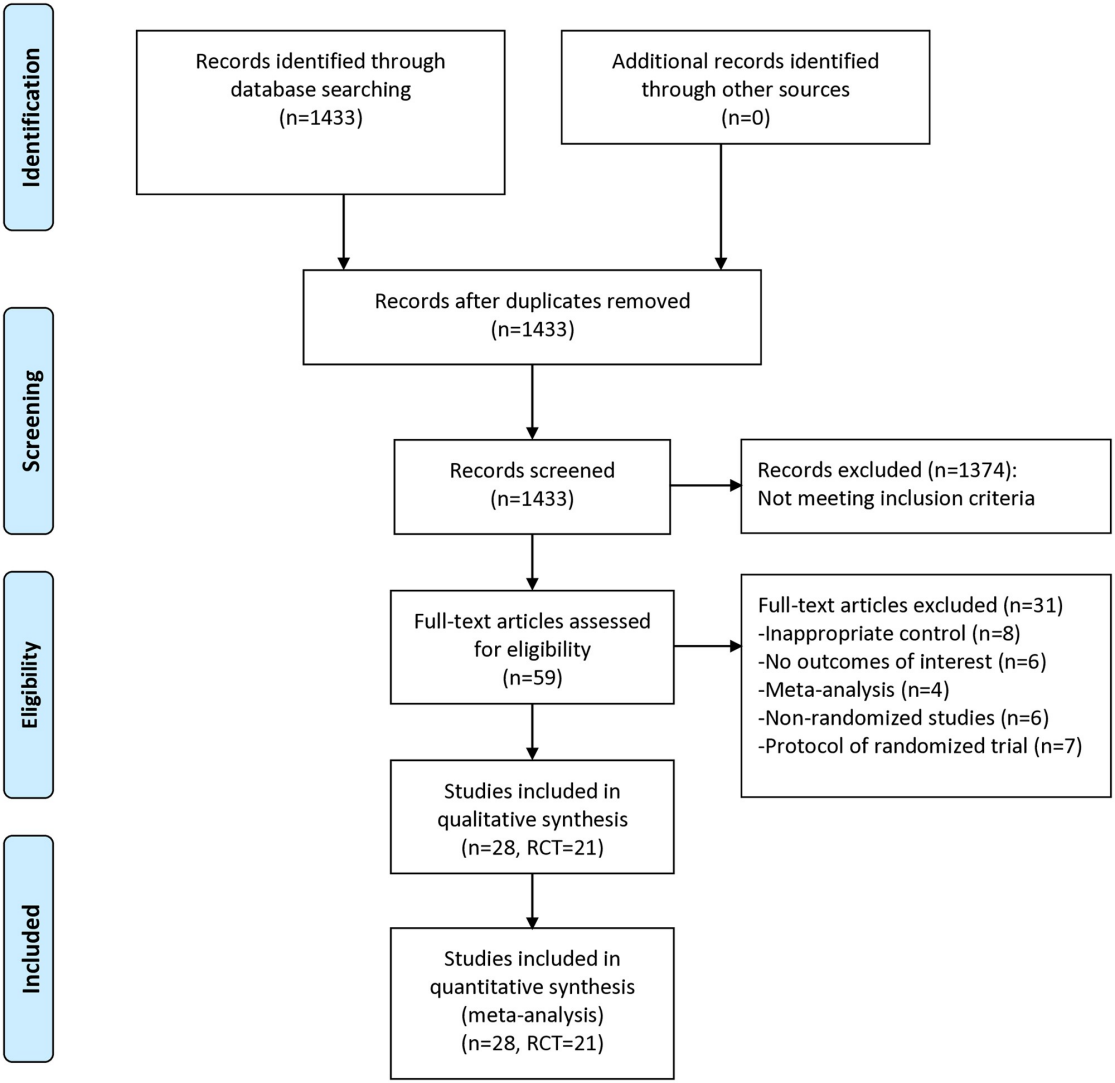
Flowchart of literature search and selection.

As shown in Fig 1, 1433 articles were retrieved from literature search, and 59 full-text articles were further reviewed for eligibility. After excluding trials with inappropriate controls (n = 8), trials without outcomes of interest (n = 6), meta-analysis (n = 4), non-randomized studies (n = 6), and protocols of randomized trials (n = 7), 28 articles reporting 21 unique RTCs were finally included for meta-analysis [12–18, 26–46] (S1 Table). A total of 1291 older adults after hip fracture surgery were assigned to home-based exercise group and 1179 patients were assigned to the control group. Five articles reported the primary or original outcomes of 5 unique RCTs [14, 16, 17, 36, 42], and 7 articles reported the long-term follow-up outcomes or secondary outcomes of interest of these 5 RCTs [33, 35, 37–39, 41, 45]. For control interventions, two trials used in-hospital rehabilitation [12, 17], two used active controls [18, 44], and the others used usual care. The time to initiate home-based exercise ranged from immediately after discharge to at average 7 months after fractures, with 13 trials initiating home exercise early after surgery (≤3 months) [12–16, 26, 29, 32, 34, 35, 40, 42, 46] and 7 initiating intervention late after surgery (>3 months) [18, 27, 28, 30, 31, 43, 44]. The home-based exercise duration of included trials varied between 1 month and 12 months, with 10 trials administering ≤3 months of intervention and 11 providing >3 months of exercise. Nine trials implemented multicomponent home-based rehabilitation programs [12, 14, 16–18, 29, 32, 35, 42], and the others applied only home exercise. Three trials had 2 arms of home-based exercise [28, 31, 36], and another trial had 2 arms of home-based exercise as well as 2 arms of control interventions [15]. The baseline characteristics of all trials included in meta-analysis were summarized in Table 1. The reported outcomes and time points of outcome assessment of each trial were listed in S2 Table. The extracted analytic data are presented in S3 and S4 Tables.

**Table 1:** Baseline characteristics of randomized trials included in the meta-analysis.

Study	Country	Sample size	Age, mean±SD	Female, N (%)	Home-based activities	Time to initiation	Control activities	Exercise duration
Sherrington, 1997	Australia	I: 20 C: 20	I: 80.0±8.1 C: 77.1±8.2	I: 12 (60.0) C: 19 (95.0)	Weight-bearing exercise	Average 7 months after fractures	Usual care	1mo
Tinetti, 1999	USA	I: 148 C: 156	I: 80.5±7.0 C: 79.4±7.8	I: 121 (71.8) C: 126 (8.08)	Strength and balance training, occupational therapy, environmental modifications	Immediately after discharge	Usual care	12mo
Hauer, 2002	Germany	I: 15 C: 13	I: 81.7±7.6 C: 80.8±7.0	I: 15 (100) C: 13 (100)	High-intensity progressive resistance training and functional training	6–8 weeks after surgery	Motor placebo activities (calisthenics, games, memory tasks whilst seated)	6mo
Crotty, 2002	Australia	I: 34 C: 32	I: 81.6 (78.2, 85.4)^#^ C: 83.5 (76.6, 85.5)^#^	I: 21 (61.8) C: 24 (75.0)	ADL-related training, environmental assessment and modifications	Average 10 days after surgery	Routine hospital care and rehabilitation	4mo
Sherrington, 2004	Australia	I1: 40 I2: 40 C: 40	I1: 80.1±7.5 I2: 79.1±8.9 C: 77.2±8.9	I1: 30 (75.0) I2: 31 (77.5) C: 34 (85.0)	I1: weight-bearing exercise I2: non-weight bearing exercise	Average 153 days after fractures	No intervention	4mo
Mangione, 2005	USA	I1: 12 I2: 11 C: 10	I1: 79.8±5.6 I2: 77.9±7.9 C: 77.8±7.3	I1: 8 (66.7) I2: 7 (63.6) C: 8 (80.0)	I1: Aerobic training I2: Resistance training	Average 19.7 weeks after surgery	Receiving biweekly mailings on a variety of nonexercise topics	12wk
Tsauo, 2005	China	I: 13 C: 12	I: 74.1±12.0 C: 71.9±12.5	I: 10 (76.9) C: 10 (83.3)	Strengthening, range-of-motion, balance, and functional exercise, practice of safe and efficient transfer techniques, adjustment of walking aids, adaption and modification of the home environment	Immediately after discharge	Continue exercise program given at bedside before discharge	3mo
Ziden, 2008	Sweden	I: 48 C: 54	I: 81.2±5.9 C: 82.5±7.6	I: 29 (60.4) C: 42 (77.8)	ADL-related training, technical aids, information about surgical treatment and prognosis, support self-efficacy	Average 1 month after discharge	Usual care	3wk
Mangione, 2010	USA	I: 14 C: 12	I: 79.6±5.9 C: 82.0±6.0	I: 12 (85.7) C: 9 (75.0)	Progressive resistance exercise	Average 6 months after fractures	Conventional TENS	10wk
Orwig, 2011	USA	I: 91 C: 89	I: 82.5±7.1 C: 82.3±6.9	I: 91 (100) C: 89 (100)	Aerobic exercise, strength training, a self-efficacy based motivational component.	Within 15 days of the fractures	Usual care	12mo
Shyu, 2013	China	I1:101 I2: 99 C: 99	I1: 76.17±6.65 I2: 76.46±7.14 C: 76.91±8.20	I1: 68 (67.3) I2: 59 (59.6) C: 64 (64.6)	I1: interdisciplinary care (geriatric consultation, rehabilitation focusing on relieving pain, enhancing range of motion, balance challenges, and aerobic capacity, discharge planning with post-hospital services) I2: comprehensive care (nutrition consultation, depression management, fall prevention, interdisciplinary care)	1st day after surgery	Usual care	12mo
Latham, 2014	USA	I: 120 C: 112	I: 77.2±10.2 C: 78.9±9.4	I: 83 (69.2) C: 77 (68.8)	Functional exercise, weight-bearing exercise	Within 24 months after fractures	Nutrition education	6mo
Salpakoski, 2014	Finland	I: 40 C: 41	I: 80.9±7.7 C: 79.1±6.4	I: 31 (77.5) C: 32 (78.0)	Promotion Mobility rehabilitation program (ProMo), including strengthening and stretching exercises, balance training, function exercises, evaluation and modification of environmental hazards, guidance for safe walking	44 to 239 days after fractures	Standard care	12mo
Karlsson, 2016	Sweden	I: 107 C: 98	I: 83.2±7.0 C: 82.6±6.4	I: 79 (73.8) C: 68 (69.4)	Functional strength and balance training, modifications of home environment, pain management, nutrition advice	NR	Conventional care and rehabilitation	10wk
Williams, 2016	UK	I: 29 C: 32	I: 80.9±6.6 C: 78.0±8.3	I: 23 (79.3) C: 23 (71.9)	Physical exercise, patient-held information workbook, goal-setting diary	Average 18.8 days after surgery	Usual care	12wk
Stemmle, 2019	Switzerland	I1: 43 I2: 44 C1: 44 C2: 42	I1: 83.2±7.4 I2: 83.5±7.1 C1: 85.5±6.0 C2: 84.6±6.9	I1: 34 (79.1) I2: 34 (77.3) C1: 35 (79.5) C2: 34 (81.0)	I1: simple home exercise program + 800 IU/d vitamin D3 I2: simple home exercise program + 2000 IU/d vitamin D3	Within 12 days after surgery	C1: Standard physiotherapy + 800 IU/d vitamin D3 C1: Standard physiotherapy + 2000 IU/d vitamin D3	12mo
Magaziner, 2019	USA	I: 105 C: 105	I: 80.3±8.0 C: 81.2±8.8	I: 80 (76.2) C: 81 (77.1)	Strength exercise, plantar flexion exercise, endurance exercise, nutritional counseling	Average 13.8w after hospitalization	Active range-of-motion exercises, sensory-level TENS	16wk
Taraldsen, 2019	Norway	I: 70 C: 73	I: 84.9±6.6 C: 82.7±5.7	I: 54 (77.1) C: 56 (76.7)	Weight-bearing exercise	Average 4 months after surgery	Usual care	10wk
Soukkio, 2021	Sweden	I: 61 C: 60	I: 83±6 C: 80±7	I: 50 (82.0) C: 41 (68.3)	Strength, balance, mobility, and function exercise; counseling on physical activity; brief advice on nutrition	Within 2 weeks of discharge from hospital	Usual care	12mo
Huang, 2023	USA	I: 17 C: 17	I: 78.6±7.3 C: 77.8±7.8	I: 7 (41.1) C: 6 (35.3)	Strength, balance, and function exercises	Average 116 days after hospitalization	Active range-of-motion exercises, TENS	16wk
Taylor, 2023	Australia	I: 20 C: 18	I: 78±9 C: 80±9	I: 10 (50.0) C: 13 (72.2)	Moderate-intensity walking intervention	Average 82 days after fractures	Standard care	12wk

^#^ Median (quartiles)

C: control group; I: intervention group; mo: months; NR: not reported; TENS: transcutaneous electrical nerve stimulation; wk: weeks.

### Risk of bias

**Fig 2:**
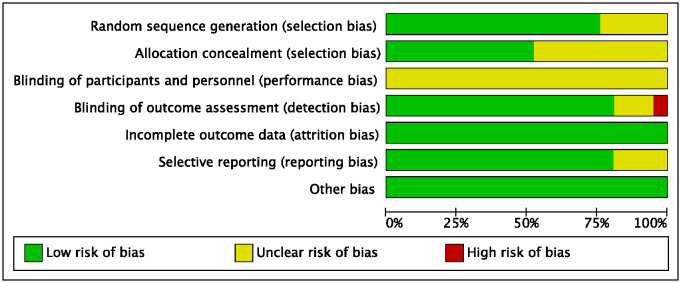
Risk of bias graph of included trials.

**Fig 3:**
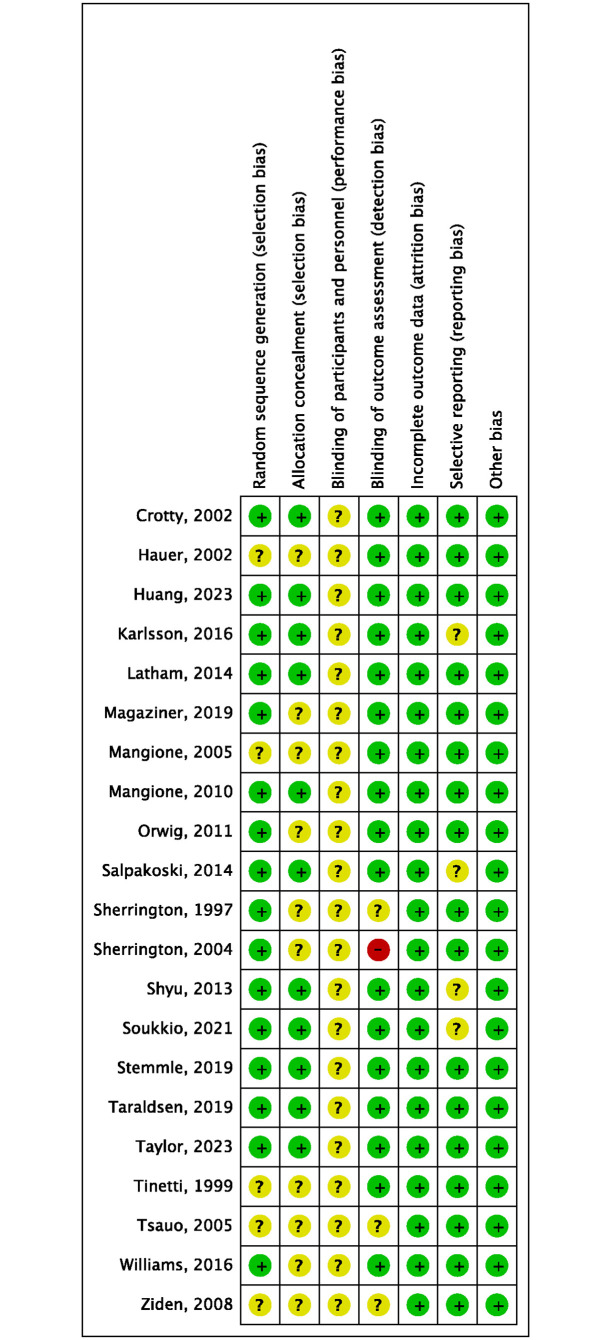
Risk of bias summary of included trials.

Since blinding of physiotherapists and patients was impossible, the bias of blinding of participants and personnel (performing bias) of all included RCTs was deemed as unclear risk. One trial reported the outcome assessors were not blinded to assignment, and therefore had high risk of detection bias [28]. Four trials were considered to have unclear risk of bias of selective reporting, as they reported the primary or original outcomes and other secondary outcomes in different articles [14, 17, 35–37, 39, 41, 42, 45]. The quality assessment of risk of bias was shown in Figs 2 and 3.

### Balance

**Fig 4:**
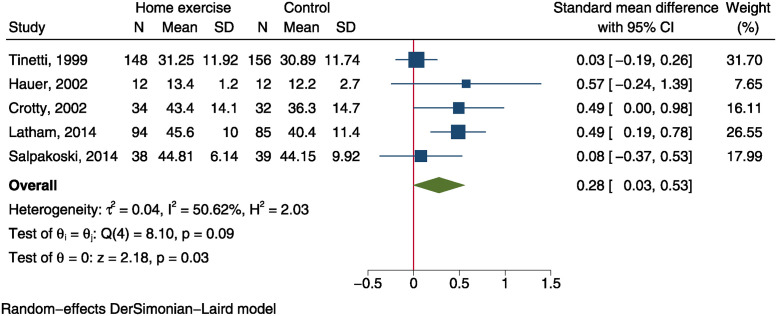
Forest plot of meta-analysis of Berg balance scale.

**Fig 5:**
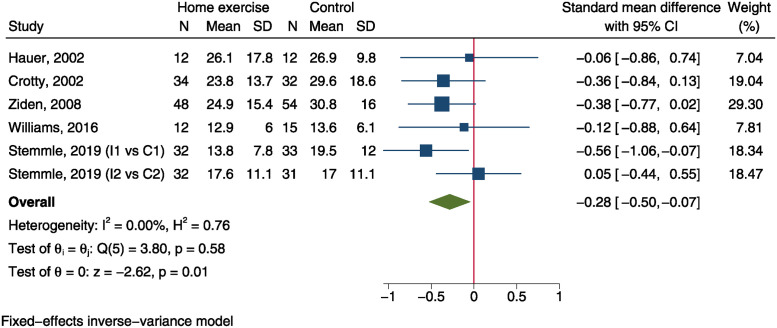
Forest plot of meta-analysis of timed-up-and-go test.

Five trials, including 326 patients in home exercise group and 324 in control group, reported BBS outcome after intervention [12–14, 26, 29]. Meta-analysis using a random-effect model demonstrated a significantly improved BBS score in home exercise group than the control group (SMD = 0.28, 95%CI: 0.03 to 0.53, P = 0.030, I^2^ = 50.6%, Fig 4). TUG was measured in 5 trials comprising 170 patients in home exercise group and 177 in control group [12, 15, 16, 26, 40]. Using a fixed-effect model, quantitative synthesis yield a SMD of -0.28 (95%CI: -0.50 to -0.07, P = 0.009, Fig 5), indicating a significantly improved balance of home exercise group than control group. However, the SMD effect sizes of both balance measurements were small.

### Mobility

**Fig 6:**
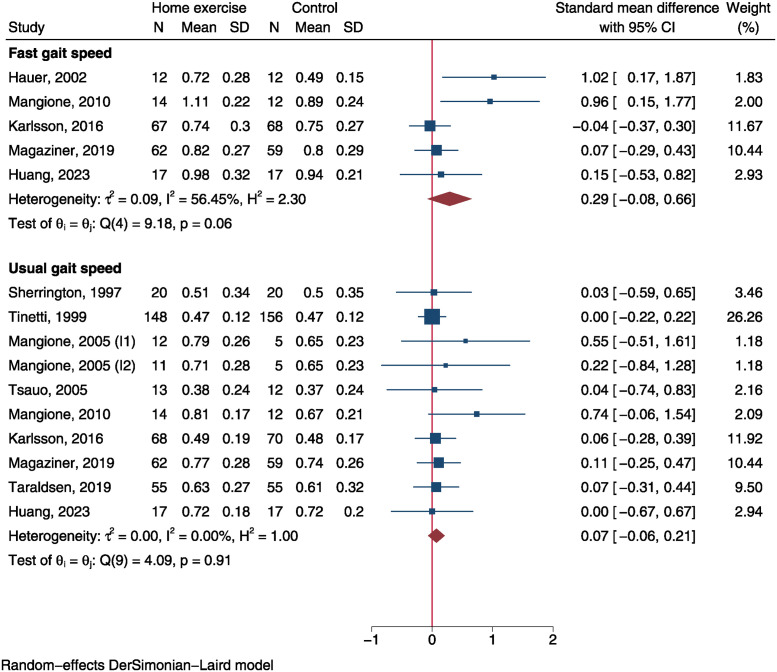
Forest plot of meta-analysis of gait speed.

Fast gait speed was measured in 5 RCTs involving 172 individuals in home exercise group and 168 in control group [17, 18, 25, 30, 44]. Usual gait speed was reported in 9 RCTs including 420 cases in home exercise group and 411 in control group [17, 18, 27, 29–31, 43, 44]. The SMD of fast gait speed was 0.29 (95%CI: -0.08 to 0.66, P = 0.120, Fig 6), which was not statistically significant. Similarly, there was no significant difference of usual gait speed between both groups (SMD = 0.07, 95%CI: -0.06 to 0.21, P = 0.302, Fig 6).

Pooled analysis of 5 trials showed no significant difference of SPPB performance between the home exercise group and the control group (SMD = 0.30, 95%CI: -0.10 to 0.69, P = 0.138, S1 Fig). Additionally, meta-analysis demonstrated home exercise did not have significantly impact on 6MWT (SMD = 0.37, 95%CI: -0.19 to 0.92, P = 0.199, S2 Fig) and walking outdoors (RR = 1.0, 95%CI: 0.83 to 1.21, P = 0.975, S3 Fig) compared to control group.

### Independence in ADL

Five RCTs including 202 fractured older adults in each group reported Barthel’s ADL, which showed no significant difference of ADL between both groups (SMD = 0.05, 95%CI: -0.29 to 0.38, P = 0.792, S4 Fig) after pooling analysis using a random-effect model. Similarly, instrumental ADL did not differ between home exercise group and control group (SMD = 0.15, 95%CI: -0.06 to 0.36, P = 0.159, S5 Fig).

### Quality of life

**Fig 7:**
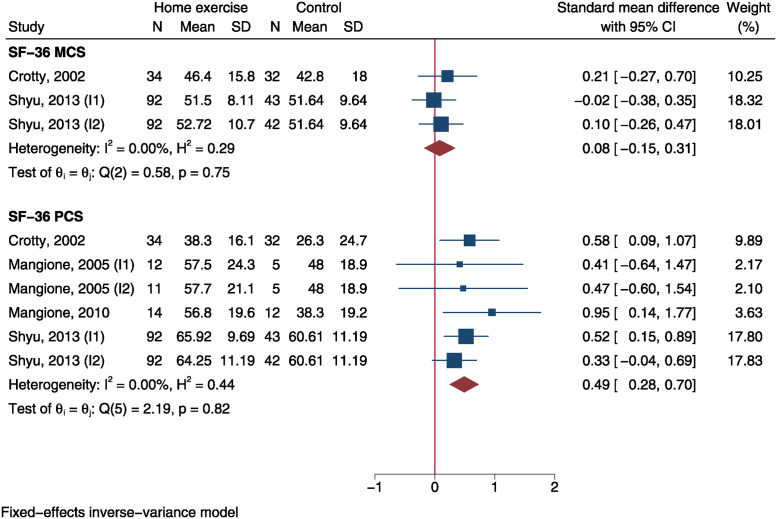
Forest plot of meta-analysis of Short Form-36 physical and mental component scores.

SF-36 PCS was measured in 4 trials and SF-36 MCS was reported in 2 trials. Using a fixed-effect model, meta-analysis demonstrated a significantly improved SF-36 PCS in home exercise group than control group (SMD = 0.49, 95%CI: 0.28 to 0.70, P<0.001) but no MCS difference between both groups (SMD = 0.08, 95%CI: -0.15 to 0.31, P = 0.486, Fig 7). The SMD effect size of SF-36 PCS was moderate.

### Muscle strength

**Fig 8:**
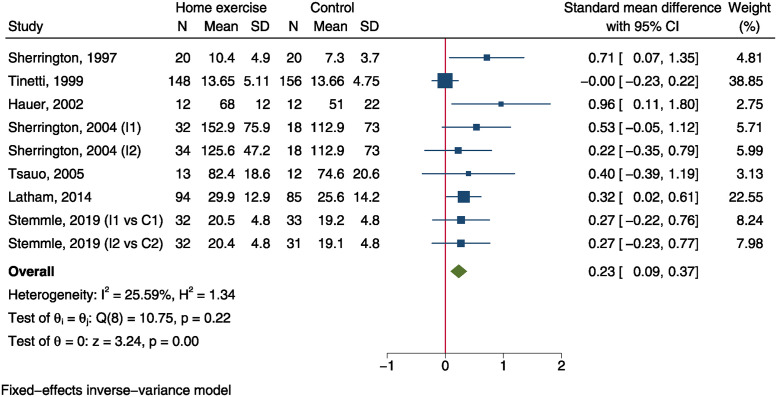
Forest plot of meta-analysis of knee extensor strength.

Knee extensor strength was measured in 7 trials [13, 15, 26–29, 32], which included 417 and 385 patients in home exercise group and control group, respectively. The SMD was 0.23 (95%CI: 0.09 to 0.37, P = 0.001, Fig 8), indicating a significantly improved strength in home exercise group than control group. However, the effect size of SMD was small.

### Fear of falling

Pooling analysis of 6 trials reporting falls efficacy scale demonstrated no significantly improved fear of falling in patients receiving home-based exercise than those in control group (SMD = 0.32, 95%CI: -0.07 to 0.71, P = 0.110, S9 Fig).

### Adverse events

There was no significant risk difference in terms of emergency department visits (RR = 0.85, 95%CI: 0.51 to 1.42, S6 Fig), hospital readmissions (RR = 0.87, 95%CI: 0.70 to 1.10, S7 Fig), and falls (RR = 0.98, 95%CI: 0.83 to 1.16, S8 Fig) between home exercise group and control group.

### Subgroup analysis

Subgroup analyses according to follow-up duration were performed to explore the short-term (≤6 months) and long-term (>6 months) effect of home-based exercise in older adults after hip fracture surgery (S5 and S6 Tables). Subgroup analysis demonstrated significant short-term impact of home exercise on TUG, falls efficacy scale, knee extensor strength, SF-36 PCS, and risk of emergency department visits. Yet, long-term impact was only observed on SF-36 PCS.

Since there were less than 10 available trials for each outcome, we did not perform meta-regression analysis to evaluate the association of intervention initiation and duration as continuous variables with the effect size. Our study revealed that early initiation of home exercise after surgery (≤3 months) was significantly associated with improved BBS, TUG, knee extensor strength and SF-36 PCS and reduced risk of hospital readmission, whereas late initiation (>3 months) was only associated with improved knee extensor strength and SF-36 PCS (S7 Table). Both short (≤3 months) and long (>3 months) duration of home exercise showed improvement in knee extensor strength and SF-36 PCS without significant between-subgroup differences (S7 Table).

Regarding rehabilitation types, exercise-only rehabilitation exhibited greater improvement in BBS (0.50 vs 0.11, P = 0.022), fast gait speed (0.63 vs 0.02, P = 0.018), and knee extensor strength (0.38 vs 0.03, P = 0.015) compared to multicomponent rehabilitation (S7 Table). On the contrary, multicomponent rehabilitation showed greater improvement in falls efficacy scale (0.91 vs 0.07, P = 0.002) and reduction in risk of emergency department visit (0.62 vs 1.52, P = 0.012) compared to exercise only (S7 Table).

### Sensitivity analysis and publication bias

Sensitivity analysis indicated the pooled effect sizes were not significantly influenced by the omission of a single study. Egger’s test showed no evidence of potential publication bias in analyses of all outcomes except fast gait speed and knee extensor strength (P = 0.007 and 0.008, respectively; S8 Table). Using a trim-and-filled method, there was still significant difference of knee extensor strength between both groups (SMD = 0.15, 95%CI: 0.02 to 0.28, S9 Fig).

### Certainty of evidence

According to GRADE approach, there was moderate certainty of evidence for outcomes of usual gait speed, SPPB, Barthel’s ADL, IADL, falls efficacy scale, and SF-35 PCS (Table 2). The overall certainty of evidence was graded as low for BBS, TUG, 6MWT, and knee extensor strength, and was very low for fast gait speed (Table 2).

**Table 2:** Assessment of the certainty of evidence using GRADE approach.

Outcomes (no. of trials)	Certainty assessment						Effect		Certainty
	Study design	Risk of bias	Inconsistency	Indirectness	Imprecision	Publication bias	No. of patients	SMD (95%CI)	
BBS (5)	RCT	Serious ^a^	Serious ^b^	Not serious	Not serious	None	326/324	0.28 (0.03, 0.53)	⊕⊕◯◯ Low
TUG (5)	RCT	Serious ^a^	Not serious	Not serious	Serious ^c^	None	170/177	-0.28 (-0.50, -0.07)	⊕⊕◯◯ Low
Fast gait speed (5)	RCT	Not serious	Serious ^b^	Not serious	Serious ^c^	Potential bias	172/168	0.29 (-0.08, 0.66)	⊕◯◯◯ Very low
Usual gait speed (10)	RCT	Serious ^a^	Not serious	Not serious	Not serious	None	420/411	0.07 (-0.07, 0.21)	⊕⊕⊕◯ Moderate
SPPB (5)	RCT	Not serious	Serious ^b^	Not serious	Not serious	None	298/289	0.30 (-0.10, 0.69)	⊕⊕⊕◯ Moderate
6MWT (4)	RCT	Not serious	Serious ^b^	Not serious	Serious ^c^	None	97/81	0.37 (-0.19, 0.92)	⊕⊕◯◯ Low
Barthel’s ADL (5)	RCT	Not serious	Serious ^b^	Not serious	Not serious	None	202/202	0.05 (-0.29, 0.38)	⊕⊕⊕◯ Moderate
IADL (5)	RCT	Not serious	Not serious	Not serious	Serious ^c^	None	172/176	0.15 (-0.06, 0.36)	⊕⊕⊕◯ Moderate
Knee extensor strength (7)	RCT	Serious ^a^	Not serious	Not serious	Not serious	Potential bias	417/385	0.23 (0.09, 0.37)	⊕⊕◯◯ Low
Falls efficacy scale (6)	RCT	Not serious	Serious ^b^	Not serious	Not serious	None	260/253	0.32 (-0.07, 0.71)	⊕⊕⊕◯ Moderate
SF-36 PCS (6)	RCT	Not serious	Not serious	Not serious	Serious ^c^	None	255/139	0.49 (0.28, 0.70)	⊕⊕⊕◯ Moderate

6MWT: 6-minute walking test; BBS: Berg balance score; GRADE: Grading of Recommendations Assessment, Development and Evaluation; IADL: instrumental activities of daily living; RCT: randomized controlled trial; SF-36 PCS: Short Form-36 questionnaire physical component score; SMD: standard mean difference; SPPB: short physical performance battery; TUG: timed-up-and-go test;

^a^ Including >25% of patients from studies with low methodological quality.

^b^ Wide variance of estimates across studies or large between-study heterogeneity (I^2^>50%)

^c^ Total number of patients <400.

## Discussion

By pooling 21 RCTs with a total sample size of 2470 older adults after hip fracture surgery, this meta-analysis demonstrates that home-based exercise, compared with control interventions, significantly improved knee extensor strength, BBS, TUG, and SF-36 PCS. GRADE assessment shows moderate certainty of evidence for SF-36 PCS and low certainty of evidence for knee extensor strength, BBS, and TUG. These results indicate that home-based exercise is effective in improving lower extremity strength, balance, mobility, and quality of life, which can be recommended for post-discharge functional rehabilitation of older adults after hip fracture surgery.

The muscle strength, balance, and mobility gradually decline in older adults [47]. Subsequent to hip fractures without timely rehabilitation, patients will rapidly lose muscle strength and physical function. Adequate skeletal muscle strength is vital for maintaining hip function and balance in postural maintenance and dynamic daily activities [48]. Post-surgery exercise training, especially those focusing on muscle strength, could significantly improve lower extremity strength, balance, and mobility [49, 50]. Higher intensity and frequency of exercise tend to be associated with greater stronger effects of functional improvement [51, 52]. Yet, whether fractured patients could benefit from home-based exercise, which is usually at a low intensity, is unclear. Our meta-analysis demonstrated a small, clinically-meaningful increase in lower extremity muscle strength (SMD = 0.23, 95%CI: 0.09 to 0.37) and balance (BBS: SMD = 0.28, 95%CI: 0.03 to 0.53; TUG: SMD = -0.28, 95%CI: -0.50, to -0.07), and a moderate, clinically-meaningful improvement in quality of life (SF-36 PCS: SMD = 0.49, 95%CI: 0.28 to 0.70) in home exercise group compared with the control group. Further analyses revealed that the improvement in quality of life were significant in both short-term and long-term assessments, suggesting that patients may persistently gain benefit for quality of life once starting home exercise. However, the benefits in improving TUG test, increasing knee extensor strength, and reducing fear of falling and risk of emergency department visit were only observed within 6-month follow-up after intervention initiation, which varnished in the long-term follow-up. More studies regarding the long-term and persistent effect of home-based exercise are needed.

The first 3 months after surgery is the best period for functional recovery, and timely initiation of rehabilitation training plays a crucial role in restoring muscle strength, improving balance, and achieving hip function [53]. In addition to benefits in muscle strength and quality of life in both early initiation (≤3 months) and late initiation (>3 months) of home exercise after surgery, our meta-analysis revealed that early initiation significantly improved balance (BBS, TUG) and reduced the risk of hospital admission compared to control interventions (S7 Table). These findings support that fractured patients will gain more benefits if they start home exercise early after surgery. The exercise duration varied greatly among included trials, and 6 RCTs implemented a yearlong home-based program [14, 15, 29, 34, 36, 42]. A 12-month individualized, multicomponent home-based rehabilitation was found to improve mobility recovery and increase physical activity after hip fractures compared with standard care, and the effect was still maintained at 1-year follow-up [14, 38, 54]. Another yearlong home-based, progressive exercise program exhibited favorable functioning and physical performance than usual care in terms of changes of IADL score, SPPB score and handgrip strength [42, 45].

The rehabilitation component is one of the major concerns affecting the functional recovery after hip fracture surgery [55, 56]. Several trials implemented a multicomponent strategy that combined home exercise with other components, such as environmental evaluation and modification, nutrition advice, pain management, and guidance for safe walking, while some only included exercise component. Multicomponent programs may enhance patient’s motivation, self-efficacy, and confidence in their ability to function in the environment in which their injury occurred [12]. As expected, subgroup analysis demonstrated that multicomponent rehabilitation exhibited greater reduction in fear of falling and risk of emergency department visit compared to exercise only. The efficacy of non-exercise components in reducing fear of falling is also observed in another multicomponent intervention, the community ageing in place, advancing better living for elders (CAPABLE), which addresses individual capacities and repairs home environment [57]. Moreover, this program is more effective in improving ADLs and IADLs [57]. Conversely, we observed greater improvements in BBS, fast gait speed, and knee extensor strength for exercise-only programs compared to multicomponent rehabilitation. However, these results need to be cautiously interpreted. Most home-based exercises in our study only applied low-intensity programs while several exercise-only programs used moderate- or high-intensity exercises. Hauer *et al*. implemented a high-intensive progressive resistance training program, which was effective to increase strength and functional performance [26]. Mangione *et al*. found high-intensity resistance training and moderate-intensity aerobic training both significantly improved low extremity force compared to control intervention [31]. Another moderate-intensity walking program increased daily physical activity [46]. Yet, the optimal exercise intensity is still debatable, as a previous study found no difference of knee extensor strength between a high-intensity exercise group and a low-intensity exercise group [58]. While usual care was used as control intervention in most trials, comparison to in-hospital rehabilitation or active control may weaken the efficacy of home-based exercise. Karlsson *et al*. observed similar results between multicomponent home-based rehabilitation and in-hospital rehabilitation in terms of gait speed and independence in ADL [17, 41]. Another trials showed comparable efficacy in BBS, TUG, and quality of life when comparing multicomponent home-based rehabilitation to in-hospital rehabilitation [12]. Magaziner *et al*., comparing multicomponent program to active control that included active range-of-motion exercises and transcutaneous electrical nerve stimulation, found no difference in 6MWT, balance, gait speed [18]. Collectively, the findings of subgroup analyses suggest that multicomponent home-based rehabilitation with more intensive exercises and initiation as early as possible after surgery may be more effective and safer for functional recovery. Future studies are needed to explore the essential rehabilitation components and optimal exercise intensity and duration for developing an effective and standardized multicomponent home-based rehabilitation program with long-lasting efficacy.

Our meta-analysis has some limitations. Firstly, the majority of included trials have low methodological quality according the risk of bias assessment. Secondly, several trials have very small sample sizes (<50), which could results in overestimated effects of home-based exercise. Thirdly, the between-study heterogeneity of several outcomes is large, which may be caused by the difference in exercise duration, intensity, frequency of home-based rehabilitation and follow-up duration. Fourthly, the exercise programs and muscles involved were highly heterogeneous across trials, making it challenging to draw a conclusion for the efficacy of a specific exercise program. Finally, the strength and balance tools varied among trials, which needs to be unified and standardized in future trials. Therefore, the results of our meta-analysis need to be cautiously interpreted.

## Conclusions

Our meta-analysis shows home-based exercise is effective in improving lower extremity strength, balance, mobility, and quality of life in older patients after hip fracture surgery. Multicomponent rehabilitation with more intensive exercises and earlier initiation can be recommended for patients after hip fracture to achieve a better functional recovery. The long-term effect and the optimal duration, intensity, frequency of home-based exercise need to be investigated in more well-designed, large-scaled RCTs.

## Supporting information

S1 Checklist PRISMA 2020 checklist. (DOCX)

S1 Table Studies for full-text review and reasons for exclusion. (DOCX)

S2 Table Reported outcomes and time points of assessment of all trials included in meta-analysis. (DOCX)

S3 Table Extracted analytic data for continuous variables. (XLSX)

S4 Table Extracted analytic data for categorical variables. (XLSX)

S5 Table Results of continuous variables for short-term and long-term effect of home exercise. (DOCX)

S6 Table Results of categorical variables for short-term and long-term effect of home exercise. (DOCX)

S7 Table Subgroup analyses according to intervention initiation time after surgery, intervention duration, and rehabilitation type. (DOCX)

S8 Table Egger’s test for publication bias. (DOCX)

S1 Fig Forest plot of meta-analysis of short physical performance battery. (PDF)

S2 Fig Forest plot of meta-analysis of 6-minte walking test. (PDF)

S3 Fig Forest plot of meta-analysis of walking outdoors. (PDF)

S4 Fig Forest plot of meta-analysis of Barthel index. (PDF)

S5 Fig Forest plot of meta-analysis of instrumental activities of daily living. (PDF)

S6 Fig Forest plot of meta-analysis of emergency department visits. (PDF)

S7 Fig Forest plot of meta-analysis of hospital readmissions. (PDF)

S8 Fig Forest plot of meta-analysis of falls. (PDF)

S9 Fig Funnel plot of knee extensor strength using a trim-and-filled method. (PDF)

## References

[1] Johansen A, Mansor M, Beck S, Mahoney H, Thomas S. Outcome following hip fracture: post-discharge residence and long-term mortality. Age Ageing. 2010;39(5):653–6. doi: 10.1093/ageing/afq074 20587442

[2] Rapp K, Becker C, Cameron ID, Klenk J, Kleiner A, Bleibler F, et al. Femoral fracture rates in people with and without disability. Age Ageing. 2012;41(5):653–8. doi: 10.1093/ageing/afs044 22431152

[3] Roth T, Kammerlander C, Gosch M, Luger TJ, Blauth M. Outcome in geriatric fracture patients and how it can be improved. Osteoporos Int. 2010;21(Suppl 4):S615–9. doi: 10.1007/s00198-010-1401-4 21058001

[4] Gullberg B, Johnell O, Kanis JA. World-wide projections for hip fracture. Osteoporos Int. 1997;7(5):407–13. doi: 10.1007/pl00004148 9425497

[5] Rapp K, Buchele G, Dreinhofer K, Bucking B, Becker C, Benzinger P. Epidemiology of hip fractures: Systematic literature review of German data and an overview of the international literature. Z Gerontol Geriatr. 2019;52(1):10–6. 29594444 10.1007/s00391-018-1382-z PMC6353815

[6] Lee H, Lee SH. Effectiveness of Multicomponent Home-Based Rehabilitation in Elderly Patients after Hip Fracture Surgery: A Randomized Controlled Trial. J Pers Med. 2022;12(4):649. doi: 10.3390/jpm12040649 35455765 PMC9027847

[7] Dyer SM, Crotty M, Fairhall N, Magaziner J, Beaupre LA, Cameron ID, et al. A critical review of the long-term disability outcomes following hip fracture. BMC Geriatr. 2016;16(1):158. doi: 10.1186/s12877-016-0332-0 27590604 PMC5010762

[8] Schaefer MS, Hammer M, Platzbecker K, Santer P, Grabitz SD, Murugappan KR, et al. What Factors Predict Adverse Discharge Disposition in Patients Older Than 60 Years Undergoing Lower-extremity Surgery? The Adverse Discharge in Older Patients after Lower-extremity Surgery (ADELES) Risk Score. Clin Orthop Relat Res. 2021;479(3):546–7. doi: 10.1097/CORR.0000000000001532 33196587 PMC7899493

[9] Hulsbaek S, Juhl C, Ropke A, Bandholm T, Kristensen MT. Exercise Therapy Is Effective at Improving Short- and Long-Term Mobility, Activities of Daily Living, and Balance in Older Patients Following Hip Fracture: A Systematic Review and Meta-Analysis. J Gerontol A Biol Sci Med Sci. 2022;77(4):861–71. doi: 10.1093/gerona/glab236 34387664

[10] Zhang X, Butts WJ, You T. Exercise interventions, physical function, and mobility after hip fracture: a systematic review and meta-analysis. Disabil Rehabil. 2022;44(18):4986–96. doi: 10.1080/09638288.2021.1924299 34101525

[11] McDonough CM, Harris-Hayes M, Kristensen MT, Overgaard JA, Herring TB, Kenny AM, et al. Physical Therapy Management of Older Adults With Hip Fracture. J Orthop Sports Phys Ther. 2021;51(2):CPG1–CPG81. doi: 10.2519/jospt.2021.0301 33522384

[12] Crotty M, Whitehead CH, Gray S, Finucane PM. Early discharge and home rehabilitation after hip fracture achieves functional improvements: a randomized controlled trial. Clin Rehabil. 2002;16(4):406–13. doi: 10.1191/0269215502cr518oa 12061475

[13] Latham NK, Harris BA, Bean JF, Heeren T, Goodyear C, Zawacki S, et al. Effect of a home-based exercise program on functional recovery following rehabilitation after hip fracture: a randomized clinical trial. JAMA. 2014;311(7):700–8. doi: 10.1001/jama.2014.469 24549550 PMC4454368

[14] Salpakoski A, Tormakangas T, Edgren J, Kallinen M, Sihvonen SE, Pesola M, et al. Effects of a multicomponent home-based physical rehabilitation program on mobility recovery after hip fracture: a randomized controlled trial. J Am Med Dir Assoc. 2014;15(5):361–8. doi: 10.1016/j.jamda.2013.12.083 24559642

[15] Stemmle J, Marzel A, Chocano-Bedoya PO, Orav EJ, Dawson-Hughes B, Freystaetter G, et al. Effect of 800 IU Versus 2000 IU Vitamin D3 With or Without a Simple Home Exercise Program on Functional Recovery After Hip Fracture: A Randomized Controlled Trial. J Am Med Dir Assoc. 2019;20(5):530–6.e1. doi: 10.1016/j.jamda.2018.10.013 30551946

[16] Ziden L, Frandin K, Kreuter M. Home rehabilitation after hip fracture. A randomized controlled study on balance confidence, physical function and everyday activities. Clin Rehabil. 2008;22(12):1019–33. doi: 10.1177/0269215508096183 19052241

[17] Karlsson A, Berggren M, Gustafson Y, Olofsson B, Lindelof N, Stenvall M. Effects of Geriatric Interdisciplinary Home Rehabilitation on Walking Ability and Length of Hospital Stay After Hip Fracture: A Randomized Controlled Trial. J Am Med Dir Assoc. 2016;17(5):464.e9–e15. 10.1016/j.jamda.2016.02.001 26975205

[18] Magaziner J, Mangione KK, Orwig D, Baumgarten M, Magder L, Terrin M, et al. Effect of a Multicomponent Home-Based Physical Therapy Intervention on Ambulation After Hip Fracture in Older Adults: The CAP Randomized Clinical Trial. JAMA. 2019;322(10):946–56. doi: 10.1001/jama.2019.12964 31503309 PMC6737521

[19] Page MJ, McKenzie JE, Bossuyt PM, Boutron I, Hoffmann TC, Mulrow CD, et al. The PRISMA 2020 statement: an updated guideline for reporting systematic reviews. BMJ. 2021;372:n71. doi: 10.1136/bmj.n71 33782057 PMC8005924

[20] Faraone SV. Interpreting estimates of treatment effects: implications for managed care. P T. 2008;33(12):700–11. 19750051 PMC2730804

[21] Lee H, Lee SH. Effectiveness of multicomponent home-based rehabilitation in older patients after hip fracture surgery: A systematic review and meta-analysis. J Clin Nurs. 2023;31(1–2):31–48. doi: 10.1111/jocn.16256 35218084

[22] Rahmati M, Yon DK, Lee SW, Udeh R, McEVoy M, Kim MS, et al. New-onset type 1 diabetes in children and adolescents as postacute sequelae of SARS-CoV-2 infection: A systematic review and meta-analysis of cohort studies. J Med Virol. 2023;95(6):e28833. doi: 10.1002/jmv.28833 37264687

[23] Rahmati M, Koyanagi A, Banitalebi E, Yon DK, Lee SW, Il Shin J, et al. The effect of SARS-CoV-2 infection on cardiac function in post-COVID-19 survivors: A systematic review and meta-analysis. J Med Virol. 2023:95(1);e28325. doi: 10.1002/jmv.28325 36401352

[24] Rahmati M, Fatemi R, Yon DK, Lee SW, Koyanagi A, Il Shin J, et al. The effect of adherence to high-quality dietary pattern on COVID-19 outcomes: A systematic review and meta-analysis. J Med Virol. 2023:95(1):e28298. doi: 10.1002/jmv.28298 36367218 PMC9877891

[25] GRADE: an emerging consensus on rating quality of evidence and strength of recommendations. BMJ. 2008;336(7650):924–6. doi: 10.1136/bmj.39489.470347.AD 18436948 PMC2335261

[26] Hauer K, Specht N, Schuler M, Bartsch P, Oster P. Intensive physical training in geriatric patients after severe falls and hip surgery. Age Ageing. 2002;31(1):49–57. doi: 10.1093/ageing/31.1.49 11850308

[27] Sherrington C, Lord SR. Home exercise to improve strength and walking velocity after hip fracture: a randomized controlled trial. Arch Phys Med Rehabil. 1997;78(2):208–12. doi: 10.1016/s0003-9993(97)90265-3 9041904

[28] Sherrington C, Lord SR, Herbert RD. A randomized controlled trial of weight-bearing versus non-weight-bearing exercise for improving physical ability after usual care for hip fracture. Arch Phys Med Rehabil. 2004;85(5):710–6. doi: 10.1016/s0003-9993(03)00620-8 15129393

[29] Tinetti ME, Baker DI, Gottschalk M, Williams CS, Pollack D, Garrett P, et al. Home-based multicomponent rehabilitation program for older persons after hip fracture: a randomized trial. Arch Phys Med Rehabil. 1999;80(8):916–22. doi: 10.1016/s0003-9993(99)90083-7 10453768

[30] Mangione KK, Craik RL, Palombaro KM, Tomlinson SS, Hofmann MT. Home-based leg-strengthening exercise improves function 1 year after hip fracture: a randomized controlled study. J Am Geriatr Soc. 2010;58(10):1911–7. doi: 10.1111/j.1532-5415.2010.03076.x 20929467 PMC2956597

[31] Mangione KK, Craik RL, Tomlinson SS, Palombaro KM. Can elderly patients who have had a hip fracture perform moderate- to high-intensity exercise at home? Phys Ther. 2005;85(8):727–39. 16048421

[32] Tsauo JY, Leu WS, Chen YT, Yang RS. Effects on function and quality of life of postoperative home-based physical therapy for patients with hip fracture. Arch Phys Med Rehabil. 2005;86(10):1953–7. doi: 10.1016/j.apmr.2005.04.020 16213237

[33] Ziden L, Kreuter M, Frandin K. Long-term effects of home rehabilitation after hip fracture—1-year follow-up of functioning, balance confidence, and health-related quality of life in elderly people. Disabil Rehabil. 2010;32(1):18–32. doi: 10.3109/09638280902980910 19925273

[34] Orwig DL, Hochberg M, Yu-Yahiro J, Resnick B, Hawkes WG, Shardell M, et al. Delivery and outcomes of a yearlong home exercise program after hip fracture: a randomized controlled trial. Arch Intern Med. 2011;171(4):323–31. doi: 10.1001/archinternmed.2011.15 21357809 PMC3140167

[35] Shyu YI, Liang J, Tseng MY, Li HJ, Wu CC, Cheng HS, et al. Comprehensive and subacute care interventions improve health-related quality of life for older patients after surgery for hip fracture: a randomised controlled trial. Int J Nurs Stud. 2013;50(8):1013–24. doi: 10.1016/j.ijnurstu.2012.11.020 23245454

[36] Shyu YI, Liang J, Tseng MY, Li HJ, Wu CC, Cheng HS, et al. Comprehensive care improves health outcomes among elderly Taiwanese patients with hip fracture. J Gerontol A Biol Sci Med Sci. 2013;68(2):188–97. doi: 10.1093/gerona/gls164 22960477

[37] Berggren M, Karlsson A, Lindelof N, Englund U, Olofsson B, Nordstrom P, et al. Effects of geriatric interdisciplinary home rehabilitation on complications and readmissions after hip fracture: a randomized controlled trial. Clin Rehabil. 2019;33(1):64–73. doi: 10.1177/0269215518791003 30064264 PMC6311618

[38] Edgren J, Salpakoski A, Sihvonen SE, Portegijs E, Kallinen M, Arkela M, et al. Effects of a home-based physical rehabilitation program on physical disability after hip fracture: a randomized controlled trial. J Am Med Dir Assoc. 2015;16(4):350.e1–7. doi: 10.1016/j.jamda.2014.12.015 25687927

[39] Shyu YI, Liang J, Tseng MY, Li HJ, Wu CC, Cheng HS, et al. Enhanced interdisciplinary care improves self-care ability and decreases emergency department visits for older Taiwanese patients over 2 years after hip-fracture surgery: A randomised controlled trial. Int J Nurs Stud. 2016;56:54–62. doi: 10.1016/j.ijnurstu.2015.12.005 26742606

[40] Williams NH, Roberts JL, Din NU, Totton N, Charles JM, Hawkes CA, et al. Fracture in the Elderly Multidisciplinary Rehabilitation (FEMuR): a phase II randomised feasibility study of a multidisciplinary rehabilitation package following hip fracture. BMJ Open. 2016;6(10):e012422. doi: 10.1136/bmjopen-2016-012422 27707828 PMC5073533

[41] Karlsson A, Lindelof N, Olofsson B, Berggren M, Gustafson Y, Nordstrom P, et al. Effects of Geriatric Interdisciplinary Home Rehabilitation on Independence in Activities of Daily Living in Older People With Hip Fracture: A Randomized Controlled Trial. Arch Phys Med Rehabil. 2020;101(4):571–8. doi: 10.1016/j.apmr.2019.12.007 31935353

[42] Soukkio PK, Suikkanen SA, Aartolahti EM, Kautiainen H, Kaaria SM, Hupli MT, et al. Effects of Home-Based Physical Exercise on Days at Home, Health Care Utilization, and Functional Independence Among Patients With Hip Fractures: A Randomized Controlled Trial. Arch Phys Med Rehabil. 2021;102(9):1692–9. doi: 10.1016/j.apmr.2021.04.004 33939973

[43] Taraldsen K, Thingstad P, Dohl O, Follestad T, Helbostad JL, Lamb SE, et al. Short and long-term clinical effectiveness and cost-effectiveness of a late-phase community-based balance and gait exercise program following hip fracture. The EVA-Hip Randomised Controlled Trial. PLoS One. 2019;14(11):e0224971. doi: 10.1371/journal.pone.0224971 31738792 PMC6860934

[44] Huang MZ, Rogers MW, Pizac D, Gruber-Baldini AL, Orwig D, Hochberg MC, et al. Effect of Multicomponent Home-Based Training on Gait and Muscle Strength in Older Adults After Hip Fracture Surgery: A Single Site Randomized Trial. Arch Phys Med Rehabil. 2023;104(2):169–78. doi: 10.1016/j.apmr.2022.08.974 36087806 PMC10039715

[45] Soukkio PK, Suikkanen SA, Kukkonen-Harjula KT, Kautiainen H, Hupli MT, Aartolahti EM, et al. Effects of a 12-month home-based exercise program on functioning after hip fracture—Secondary analyses of an RCT. J Am Geriatr Soc. 2022;70(9):2561–70. doi: 10.1111/jgs.17824 35582993 PMC9790677

[46] Taylor NF, Shields N, Thompson AL, O’Halloran PD, Harding KE, Dennett AM, et al. A walking programme for adults living in the community after hip fracture: A feasibility randomized controlled trial. Clin Rehabil. 2023;37(1):47–59. doi: 10.1177/02692155221128721 36163694

[47] Nakano MM, Otonari TS, Takara KS, Carmo CM, Tanaka C. Physical performance, balance, mobility, and muscle strength decline at different rates in elderly people. J Phys Ther Sci. 2014;26(4):583–6. doi: 10.1589/jpts.26.583 24764638 PMC3996426

[48] Mard M, Vaha J, Heinonen A, Portegijs E, Sakari-Rantala R, Kallinen M, et al. The effects of muscle strength and power training on mobility among older hip fracture patients. Adv Physiother. 2008;10:195–202.

[49] Pan RJ, Gui SJ, He YL, Nian F, Ni XY, Zhou YH, et al. The effectiveness of optimal exercise-based strategy for patients with hip fracture: a systematic review and Bayesian network meta-analysis. Sci Rep. 2023;13(1):10521. doi: 10.1038/s41598-023-37509-y 37386114 PMC10310779

[50] Ramadi A, Ezeugwu VE, Weber S, Funabashi M, Lima CA, Perracini MR, et al. Progressive Resistance Training Program Characteristics in Rehabilitation Programs Following Hip Fracture: A Meta-Analysis and Meta-Regression. Geriatr Orthop Surg Rehabil. 2022;13:21514593221090799. doi: 10.1177/21514593221090799 35514534 PMC9067046

[51] Bai F, Leng M, Zhang Y, Guo J, Wang Z. Effectiveness of intensive versus regular or no exercise in older adults after hip fracture surgery: A systematic review and meta-analysis. Braz J Phys Ther. 2023;27(1):100482. doi: 10.1016/j.bjpt.2023.100482 36738661 PMC9932354

[52] Fairhall NJ, Dyer SM, Mak JC, Diong J, Kwok WS, Sherrington C. Interventions for improving mobility after hip fracture surgery in adults. Cochrane Database Syst Rev. 2022;9(9):CD001704. doi: 10.1002/14651858.CD001704.pub5 36070134 PMC9451000

[53] Chen B, Hu N, Tan JH. Efficacy of home-based exercise programme on physical function after hip fracture: a systematic review and meta-analysis of randomised controlled trials. Int Wound J. 2020;17(1);45–54. doi: 10.1111/iwj.13230 31714005 PMC7948621

[54] Turunen K, Salpakoski A, Edgren J, Tormakangas T, Arkela M, Kallinen M, et al. Physical Activity After a Hip Fracture: Effect of a Multicomponent Home-Based Rehabilitation Program-A Secondary Analysis of a Randomized Controlled Trial. Arch Phys Med Rehabil. 2017;98(5):981–8. doi: 10.1016/j.apmr.2017.01.004 28137475

[55] Auais MA, Eilayyan O, Mayo NE. Extended exercise rehabilitation after hip fracture improves patients’ physical function: a systematic review and meta-analysis. Phys Ther. 2012;92(11):1437–51. doi: 10.2522/ptj.20110274 22822235

[56] Handoll HH, Sherrington C, Mak JC. Interventions for improving mobility after hip fracture surgery in adults. Cochrane Database Syst Rev. 2011;(3):CD001704. doi: 10.1002/14651858.CD001704.pub4 21412873

[57] Rahmati M, Keshvari M, Koyanagi A, Yong DK, Lee SW, Il Shin J, et al. The effectiveness of community ageing in place, advancing better living for elders as a biobehavioural environmental approach for disability among low-income older adults: a systematic review and meta-analysis. Age Ageing. 2023;52(4):afad053. doi: 10.1093/ageing/afad053 37078754

[58] Moseley AM, Sherrington C, Lord SR, Barraclough E, St George RJ, Cameron ID. Mobility training after hip fracture: a randomised controlled trial. Age Ageing. 2009;38(1):74–80. doi: 10.1093/ageing/afn217 19017676

