# Supplementary material for: Effectiveness of home-based exercise for functional rehabilitation in older adults after hip fracture surgery: A systematic review and meta-analysis of randomized controlled trials
Source: PLoS One. 2024 Dec 19;19(12):e0315707. doi: 10.1371/journal.pone.0315707 (PMC11658508; doi:10.1371/journal.pone.0315707)
Supplement: S1 Table — (DOCX) [file pone.0315707.s002.docx]

S1 Table. Studies for full-text review and reasons for exclusion.

| No. | Title | DOI | Eligibility |
| --- | --- | --- | --- |
| 1 | Home exercise to improve strength and walking velocity after hip fracture: a randomized controlled trial. | 10.1016/s0003-9993(97)90265-3 | Included |
| 2 | Home-based multicomponent rehabilitation program for older persons after hip fracture: a randomized trial | 10.1016/s0003-9993(99)90083-7 | Included |
| 3 | Intensive physical training in geriatric patients after severe falls and hip surgery. | 10.1093/ageing/31.1.49 | Included |
| 4 | Early discharge and home rehabilitation after hip fracture achieves functional improvements: a randomized controlled trial. | 10.1191/0269215502cr518oa | Included |
| 5 | A randomized controlled trial of weight-bearing versus non-weight-bearing exercise for improving physical ability after usual care for hip fracture. | 10.1016/s0003-9993(03)00620-8 | Included |
| 6 | Can elderly patients who have had a hip fracture perform moderate- to high-intensity exercise at home? | https://pubmed.ncbi.nlm.nih. gov/16048421/ | Included |
| 7 | Effects on function and quality of life of postoperative home-based physical therapy for patients with hip fracture. | 10.1016/j.apmr.2005.04.020 | Included |
| 8 | Home rehabilitation after hip fracture. A randomized controlled study on balance confidence, physical function and everyday activities. | 10.1177/0269215508096183 | Included |
| 9 | Long-term effects of home rehabilitation after hip fracture - 1-year follow-up of functioning, balance confidence, and health-related quality of life in elderly people. | 10.3109/09638280902980910 |  |
| 10 | Home-based leg-strengthening exercise improves function 1 year after hip fracture: a randomized controlled study. | 10.1111/j.1532-5415.2010.03076.x | Included |
| 11 | Delivery and outcomes of a yearlong home exercise program after hip fracture: a randomized controlled trial. | - 10.1001/archinternmed.2011.15 | Included |
| 12 | Comprehensive and subacute care interventions improve health-related quality of life for older patients after surgery for hip fracture: a randomised controlled trial. | 10.1016/j.ijnurstu.2012.11.020 | Included |
| 13 | Comprehensive care improves health outcomes among elderly Taiwanese patients with hip fracture. | 10.1093/gerona/gls164 | Included |
| 14 | Effect of a home-based exercise program on functional recovery following rehabilitation after hip fracture: a randomized clinical trial. | 10.1001/jama.2014.469 | Included |
| 15 | Effects of a multicomponent home-based physical rehabilitation program on mobility recovery after hip fracture: a randomized controlled trial. | 10.1016/j.jamda.2013.12.083 | Included |
| 16 | Effects of a home-based physical rehabilitation program on physical disability after hip fracture: a randomized controlled trial. | 10.1016/j.jamda.2014.12.015 |  |
| 17 | Enhanced interdisciplinary care improves self-care ability and decreases emergency department visits for older Taiwanese patients over 2 years after hip-fracture surgery: A randomised controlled trial. | 10.1016/j.ijnurstu.2015.12.005 |  |
| 18 | Effects of Geriatric Interdisciplinary Home Rehabilitation on Walking Ability and Length of Hospital Stay After Hip Fracture: A Randomized Controlled Trial. | 10.1016/j.jamda.2016.02.001 | Included |
| 19 | Fracture in the Elderly Multidisciplinary Rehabilitation (FEMuR): a phase II randomised feasibility study of a multidisciplinary rehabilitation package following hip fracture. | 10.1136/bmjopen-2016-012422 | Included |
| 20 | Effects of geriatric interdisciplinary home rehabilitation on complications and readmissions after hip fracture: a randomized controlled trial. | 10.1177/0269215518791003 |  |
| 21 | Effect of 800 IU Versus 2000 IU Vitamin D3 With or Without a Simple Home Exercise Program on Functional Recovery After Hip Fracture: A Randomized Controlled Trial. | 10.1016/j.jamda.2018.10.013 | Included |
| 22 | Effect of a Multicomponent Home-Based Physical Therapy Intervention on Ambulation After Hip Fracture in Older Adults: The CAP Randomized Clinical Trial. | 10.1001/jama.2019.12964 | Included |
| 23 | Short and long-term clinical effectiveness and cost-effectiveness of a late-phase community-based balance and gait exercise program following hip fracture. The EVA-Hip Randomised Controlled Trial. | 10.1371/journal.pone.0224971 | Included |
| 24 | Effects of Geriatric Interdisciplinary Home Rehabilitation on Independence in Activities of Daily Living in Older People With Hip Fracture: A Randomized Controlled Trial. | 10.1016/j.apmr.2019.12.007 |  |
| 25 | Effects of Home-Based Physical Exercise on Days at Home, Health Care Utilization, and Functional Independence Among Patients With Hip Fractures: A Randomized Controlled Trial. | 10.1016/j.apmr.2021.04.004 | Included |
| 26 | Effects of a 12-month home-based exercise program on functioning after hip fracture - Secondary analyses of an RCT. | 10.1111/jgs.17824 |  |
| 27 | Effect of Multicomponent Home-Based Training on Gait and Muscle Strength in Older Adults After Hip Fracture Surgery: A Single Site Randomized Trial. | 10.1016/j.apmr.2022.08.974 | Included |
| 28 | A walking programme for adults living in the community after hip fracture: A feasibility randomized controlled trial. | 10.1177/02692155221128721 | Included |
| 29 | A randomized, controlled comparison of home versus institutional rehabilitation of patients with hip fracture. | 10.1191/0269215502cr525oa | Excluded (trials without outcomes of interest) |
| 30 | Patient and caregiver outcomes 12 months after home-based therapy for hip fracture: a randomized controlled trial. | 10.1016/s0003-9993(03)00141-2 | Excluded (trials without outcomes of interest) |
| 31 | Effects of extended outpatient rehabilitation after hip fracture: a randomized controlled trial. | 10.1001/jama.292.7.837 | Excluded (inappropriate control) |
| 32 | Multi-centre cluster randomised trial comparing a community group exercise programme with home based exercise with usual care for people aged 65 and over in primary care: protocol of the ProAct 65+ trial. | 10.1186/1745-6215-11-6 | Excluded (protocol) |
| 33 | Home based exercise to improve turning and mobility performance among community dwelling older adults: protocol for a randomized controlled trial. | 10.1186/1471-2318-14-100 | Excluded (protocol) |
| 34 | The effects of habitual functional training on physical functioning in patients after hip fracture: the protocol of the HIPFRAC study. | 10.1186/s12877-016-0398-8 | Excluded (protocol) |
| 35 | Physical Activity After a Hip Fracture: Effect of a Multicomponent Home-Based Rehabilitation Program-A Secondary Analysis of a Randomized Controlled Trial. | 10.1016/j.apmr.2017.01.004 | Excluded (trials without outcomes of interest) |
| 36 | Effect of home-based rehabilitation for hip fracture: A meta-analysis of randomized controlled trials. | 10.2340/16501977-2328 | Excluded (meta-analysis) |
| 37 | Effectiveness of Supervised Home-Based Exercise Therapy Compared to a Control Intervention on Functions, Activities, and Participation in Older Patients After Hip Fracture: A Systematic Review and Meta-analysis. | 10.1016/j.apmr.2018.05.006 | Excluded (meta-analysis) |
| 38 | Effects of 12-month home-based physiotherapy on duration of living at home and functional capacity among older persons with signs of frailty or with a recent hip fracture - protocol of a randomized controlled trial (HIPFRA study) | - 10.1186/s12877-018-0916-y | Excluded (protocol) |
| 39 | Effectiveness of home based intervention program in reducing mortality of hip fracture patients: A non-randomized controlled trial | 10.1016/j.archger.2018.11.007 | Excluded (non-RCT) |
| 40 | Multifactorial intervention for hip and pelvic fracture patients with mild to moderate cognitive impairment: study protocol of a dual-centre randomised controlled trial (OF-CARE) | 10.1186/s12877-019-1133-z | Excluded (protocol) |
| 41 | Effects of a Home-Based Physical Rehabilitation Program on Tibial Bone Structure, Density, and Strength After Hip Fracture: A Secondary Analysis of a Randomized Controlled Trial | 10.1002/jbm4.10175 | Excluded (trials without outcomes of interest) |
| 42 | Efficacy of home-based exercise programme on physical function after hip fracture: a systematic review and meta-analysis of randomised controlled trials | 10.1111/iwj.13230 | Excluded (meta-analysis) |
| 43 | Effects of home-based occupational therapy telerehabilitation via smartphone for outpatients after hip fracture surgery: A feasibility randomised controlled study | 10.1177/1357633X20932434 | Excluded (inappropriate control) |
| 44 | Effectiveness of home-based rehabilitation program in minimizing disability and secondary falls after a hip fracture: Protocol for a randomized controlled trial | 10.1016/j.isjp.2020.06.002 | Excluded (protocol) |
| 45 | Geriatric Interdisciplinary Home Rehabilitation After Hip Fracture in People with Dementia - A Subgroup Analysis of a Randomized Controlled Trial | 10.2147/CIA.S250809 | Excluded (trials without outcomes of interest) |
| 46 | Intensive Inpatient vs. Home-Based Rehabilitation After Hip Fracture in the Elderly Population | 10.3389/fmed.2020.592693 | Excluded (non-RCT) |
| 47 | Effects of exercise training on bone mineral density and some health-related outcomes in HIV conditions: A randomized controlled trial | - 10.1097/MD.0000000000023206 | Excluded (protocol) |
| 48 | Effectiveness of a Home-Based Fragility Fracture Integrated Rehabilitation Management (FIRM) Program in Patients Surgically Treated for Hip Fractures | 10.3390/jcm10010018 | Excluded (non-RCT) |
| 49 | Effects of Tele-Rehabilitation Compared with Home-Based in-Person Rehabilitation for Older Adult's Function after Hip Fracture | 10.3390/ijerph18105493 | Excluded (inappropriate control) |
| 50 | Outpatient and Home-Based Treatment: Effective Settings for Hip Fracture Rehabilitation in Elderly Patients | 10.3390/geriatrics6030083 | Excluded (non-RCT) |
| 51 | Effects of the @ctivehip telerehabilitation program on the quality of life, psychological factors and fitness level of patients with hip fracture | 10.1177/1357633X211073256 | Excluded (non-RCT) |
| 52 | Effectiveness of multicomponent home-based rehabilitation in older patients after hip fracture surgery: A systematic review and meta-analysis | 10.1111/jocn.16256 | Excluded (meta-analysis) |
| 53 | Effectiveness of Multicomponent Home-Based Rehabilitation in Elderly Patients after Hip Fracture Surgery: A Randomized Controlled Trial | 10.3390/jpm12040649 | Excluded (inappropriate control) |
| 54 | Effect of Home-based Telerehabilitation on the Postoperative Rehabilitation Outcome of Hip Fracture in the Aging Population | 10.1111/os.13293 | Excluded (inappropriate control) |
| 55 | Clinical Effectiveness of Home-Based Telerehabilitation Program for Geriatric Hip Fracture Following Total Hip Replacement | 10.1111/os.13521 | Excluded (inappropriate control) |
| 56 | Implementing an intervention to enhance care delivery and consistency for people with hip fracture and cognitive impairment in acute hospital wards: a mixed methods process evaluation of a randomised controlled feasibility trial (PERFECTED) | 10.1136/bmjopen-2022-064482 | Excluded (trials without outcomes of interest) |
| 57 | Rehabilitation Outcomes Following Hip Fracture of Home-Based Exercise Interventions Using a Wearable Device-A Randomized Controlled Pilot and Feasibility Study | 10.3390/ijerph20043107 | Excluded (inappropriate control) |
| 58 | Use of mobile app to enhance functional outcomes and adherence of home-based rehabilitation program for elderly with hip fracture: A randomized controlled trial | 10.1142/S101370252250010X | Excluded (inappropriate control) |
| 59 | Multicomponent Home-based Physical Therapy Versus Usual Care for Recovery After Hip Fracture | 10.1016/j.apmr.2023.05.001 | Excluded (non-RCT) |
