# Supplementary material for: Effectiveness of home-based exercise for functional rehabilitation in older adults after hip fracture surgery: A systematic review and meta-analysis of randomized controlled trials
Source: PLoS One. 2024 Dec 19;19(12):e0315707. doi: 10.1371/journal.pone.0315707 (PMC11658508; doi:10.1371/journal.pone.0315707)
Supplement: S2 Table — (DOCX) [file pone.0315707.s003.docx]

S2 Table. Reported outcomes and time points of assessment of all trials included in meta-analysis.

| Study | 1 | 2 | 3 | 4 | 5 | 6 | 7 | 8 | 9 | 10 | 11 | 12 | 13 | 14 | 15 | 16 | Assessment time |
| --- | --- | --- | --- | --- | --- | --- | --- | --- | --- | --- | --- | --- | --- | --- | --- | --- | --- |
| Sherrington, 1997 |  |  |  |  |  |  | 🗸 |  |  | 🗸 |  |  |  |  |  |  | 1mo |
| Tinetti, 1999 | 🗸 |  |  |  |  |  | 🗸 |  |  | 🗸 |  |  |  |  |  |  | 6mo, 12mo |
| Hauer, 2002 | 🗸 | 🗸 | 🗸 | 🗸 |  | 🗸 |  |  |  | 🗸 |  |  |  |  |  |  | 6mo |
| Crotty, 2002 | 🗸 | 🗸 | 🗸 |  |  |  |  |  | 🗸 |  | 🗸 | 🗸 |  | 🗸 | 🗸 |  | 4mo |
| Sherrington, 2004 |  |  |  |  |  |  |  |  |  | 🗸 |  |  |  |  |  |  | 4mo |
| Mangione, 2005 |  |  |  |  |  |  | 🗸 | 🗸 |  |  | 🗸 |  |  |  |  |  | 12wk |
| Tsauo, 2005 |  |  |  |  |  |  |  |  |  | 🗸 |  |  |  |  |  |  | 6mo |
| Ziden, 2008 |  | 🗸 |  |  |  |  |  |  | 🗸 |  |  |  |  | 🗸 |  | 🗸 | 6mo, 12mo |
| Mangione, 2010 |  |  |  |  |  | 🗸 | 🗸 | 🗸 |  |  | 🗸 |  |  |  |  |  | 12mo |
| Orwig, 2011 |  |  |  |  |  |  |  |  |  |  |  |  | 🗸 | 🗸 | 🗸 |  | 12mo |
| Shyu, 2013 |  |  |  |  |  |  |  |  |  |  | 🗸 | 🗸 | 🗸 | 🗸 | 🗸 |  | 6mo, 12mo |
| Latham, 2014 | 🗸 |  |  |  | 🗸 |  |  |  | 🗸 | 🗸 |  |  |  |  |  |  | 6mo, 9mo |
| Salpakoski, 2014 | 🗸 |  |  | 🗸 | 🗸 |  |  |  |  |  |  |  |  |  |  |  | 6mo, 12mo |
| Karlsson, 2016 |  |  | 🗸 |  |  | 🗸 | 🗸 |  |  |  |  |  |  | 🗸 | 🗸 | 🗸 | 3mo, 12mo |
| Williams, 2016 |  | 🗸 | 🗸 | 🗸 |  |  |  |  | 🗸 |  |  |  |  |  |  |  | 12wk |
| Stemmle, 2019 |  | 🗸 |  |  |  |  |  |  |  | 🗸 |  |  |  |  |  |  | 12mo |
| Magaziner, 2019 |  |  |  |  | 🗸 | 🗸 | 🗸 | 🗸 |  |  |  |  |  | 🗸 |  | 🗸 | 16wk, 40wk |
| Taraldsen, 2019 |  |  | 🗸 | 🗸 | 🗸 |  |  |  | 🗸 |  |  |  |  |  |  | 🗸 | 6mo, 12mo |
| Soukkio, 2021 |  |  |  | 🗸 | 🗸 |  |  |  |  |  |  |  |  |  |  |  | 12mo |
| Huang, 2023 |  |  |  |  |  | 🗸 | 🗸 |  |  |  |  |  |  |  |  |  | 16wk |
| Taylor, 2023 |  |  |  |  |  |  |  |  | 🗸 |  |  |  | 🗸 | 🗸 | 🗸 |  | 12wk |

1: Berg balance score;

2: Timed-up-and-go test;

3: Barthel's ADL;

4: Instrumental ADL;

5: Short physical performance battery;

6: Fast gait speed;

7: Usual gait speed;

8: 6-minute walking test;

9: Falls efficacy scale;

10: Knee extensor strength;

11: Short Form 36 questionnaire physical component score;

12: Short-Form 36 questionnaire mental component score;

13: Emergency department visit;

14: Falls;

15: Hospital readmission;

16: Walking outdoors.

ADL: activities of daily living; mo: months; wk: weeks.
