# Supplementary material for: Effectiveness of home-based exercise for functional rehabilitation in older adults after hip fracture surgery: A systematic review and meta-analysis of randomized controlled trials
Source: PLoS One. 2024 Dec 19;19(12):e0315707. doi: 10.1371/journal.pone.0315707 (PMC11658508; doi:10.1371/journal.pone.0315707)
Supplement: S5 Table — (DOCX) [file pone.0315707.s006.docx]

S5 Table. Results of continuous variables for short-term and long-term effect of home exercise.

| Outcomes | No. | Sample | I^2^ (%) | Model | SMD (95%CI) | P |
| --- | --- | --- | --- | --- | --- | --- |
| Short-term (≤6 months) |  |  |  |  |  |  |
| Berg balance score | 5 | 333/334 | 49.0 | Random | 0.17 (-0.07, 0.42) | 0.157 |
| Timed-up-and-go test | 4 | 106/113 | 0 | Fixed | **-0.30 (-0.57, -0.04)** | **0.027** |
| Barthel's ADL | 5 | 223/217 | 63.1 | Random | 0.07 (-0.33, 0.35) | 0.966 |
| Instrumental ADL | 4 | 131/133 | 0 | Fixed | 0.16 (-0.09, 0.40) | 0.190 |
| SPPB | 4 | 291/291 | 52.4 | Random | 0.10 (-0.15, 0.34) | 0.435 |
| Fast gait speed | 4 | 195/194 | 41.6 | Fixed | 0.08 (-0.12, 0.28) | 0.420 |
| Usual gait speed | 9 | 451/447 | 0 | Fixed | 0.09 (-0.05, 0.22) | 0.202 |
| 6MWT | 3 | 114/106 | 0 | Fixed | 0.14 (-0.13, 0.41) | 0.302 |
| Falls efficacy scale | 6 | 278/265 | 56.3 | Random | **0.29 (0.02, 0.57)** | **0.038** |
| Knee extensor strength | 7 | 359/331 | 51.6 | Random | **0.27 (0.01, 0.53)** | **0.040** |
| SF-36 PCS | 5 | 246/134 | 0 | Fixed | **0.31 (0.09, 0.52)** | **0.005** |
| SF-36 MCS | 3 | 223/124 | 0 | Fixed | 0.03 (-0.19, 0.25) | 0.807 |
| Long-term (>6 months) |  |  |  |  |  |  |
| Berg balance score | 3 | 280/280 | 66.5 | Random | 0.20 (-0.11, 0.51) | 0.204 |
| Timed-up-and-go test | 2 | 64/64 | 66.4 | Random | -0.25 (-0.86, 0.35) | 0.410 |
| Barthel's ADL | 2 | 135/136 | 23.1 | Fixed | -0.12 (-0.36, 0.12) | 0.341 |
| Instrumental ADL | 3 | 140/144 | 0 | Fixed | 0.12 (-0.11, 0.36) | 0.304 |
| SPPB | 5 | 298/289 | 82.2 | Random | 0.30 (-0.10, 0.69) | 0.138 |
| Fast gait speed | 3 | 143/139 | 59.3 | Random | 0.18 (-0.22, 0.59) | 0.379 |
| Usual gait speed | 5 | 347/352 | 0 | Fixed | 0.07 (-0.08, 0.22) | 0.380 |
| 6MWT | 2 | 74/71 | 82.6 | Random | 0.46 (-0.61, 1.53) | 0.400 |
| Falls efficacy scale | 3 | 187/187 | 87.9 | Random | 0.43 (-0.19, 1.04) | 0.176 |
| Knee extensor strength | 4 | 306/305 | 13.3 | Fixed | 0.15 (-0.01, 0.31) | 0.069 |
| SF-36 PCS | 3 | 198/97 | 0 | Fixed | **0.47 (0.23, 0.72)** | **<0.001** |
| SF-36 MCS | 3 | 223/124 | 0 | Fixed | 0.04 (-0.21, 0.30) | 0.741 |

6MWT: 6-minute walking test; ADL: activities of daily living; MCS: mental component score; PCS: physical component score; SF-36: Short Form-36 questionnaire; SMD: standard mean difference; SPPB: short physical performance battery.
