# Supplementary material for: Effectiveness of home-based exercise for functional rehabilitation in older adults after hip fracture surgery: A systematic review and meta-analysis of randomized controlled trials
Source: PLoS One. 2024 Dec 19;19(12):e0315707. doi: 10.1371/journal.pone.0315707 (PMC11658508; doi:10.1371/journal.pone.0315707)
Supplement: S6 Table — (DOCX) [file pone.0315707.s007.docx]

S6 Table. Results of categorical variables for short-term and long-term effect of home exercise.

| Outcomes | No. | Sample | I^2^ (%) | Model | RR (95%CI) | P |
| --- | --- | --- | --- | --- | --- | --- |
| Short-term |  |  |  |  |  |  |
| Emergency department visit | 3 | 219/106 | 46.9 | Fixed | **0.56 (0.35-0.90)** | **0.016** |
| Falls | 5 | 355/231 | 5.7 | Fixed | 0.90 (0.68-1.19) | 0.473 |
| Hospital readmission | 5 | 355/231 | 0 | Fixed | 0.78 (0.54-1.12) | 0.174 |
| Walking outdoors | 4 | 294/300 | 72.9 | Random | 1.18 (0.77-1.82) | 0.456 |
| Long-term |  |  |  |  |  |  |
| Emergency department visit | 3 | 291/178 | 52.1 | Random | 0.78 (0.48-1.25) | 0.297 |
| Falls | 6 | 547/425 | 9.7 | Fixed | 0.96 (0.81-1.13) | 0.597 |
| Hospital readmission | 4 | 397/271 | 24.8 | Fixed | 0.90 (0.72-1.13) | 0.369 |
| Walking outdoors7 | 3 | 196/191 | 0 | Fixed | 1.06 (0.87-1.28) | 0.573 |

RR: risk ratio.
