# Supplementary material for: Effectiveness of home-based exercise for functional rehabilitation in older adults after hip fracture surgery: A systematic review and meta-analysis of randomized controlled trials
Source: PLoS One. 2024 Dec 19;19(12):e0315707. doi: 10.1371/journal.pone.0315707 (PMC11658508; doi:10.1371/journal.pone.0315707)
Supplement: S7 Table — (DOCX) [file pone.0315707.s008.docx]

S7 Table. Subgroup analyses according to intervention initiation time after surgery, intervention duration, and rehabilitation type.

| Outcomes | No. of studies | I^2^ (%) | Model | Effect size (95%CI) | P | P ^#^ |
| --- | --- | --- | --- | --- | --- | --- |
| **Berg Balance Score** | | | | SMD |  |  |
| Intervention initiation time after surgery (months) | | | |  |  | - |
| ≤3 | 5 | 50.6 | Random | 0.28 (0.03, 0.53) | **0.030** |  |
| >3 | 0 | - | - | - | - |  |
| Intervention duration (months) | | | |  |  | - |
| ≤3 | 1 | - | - | - | - |  |
| >3 | 4 | 59.3 | Random | 0.25 (-0.02, 0.52) | 0.066 |  |
| Rehabilitation type |  |  |  |  |  | **0.022** |
| Multicomponent | 3 | 29.7 | Fixed | 0.11 (-0.08, 0.29) | 0.266 |  |
| Exercise only | 2 | 0 | Fixed | 0.50 (0.22, 0.78) | **<0.001** |  |
| **Timed-up-and-go test** | | | | SMD |  |  |
| Intervention initiation time after surgery (months) | | | |  |  | - |
| ≤3 | 6 | 0 | Fixed | -0.26 (-0.50, -0.07) | **0.009** |  |
| >3 | 0 | - | - | - | - |  |
| Intervention duration (months) | | | |  |  | 0.965 |
| ≤3 | 3 | 0 | Fixed | -0.28 (-0.60, 0.04) | 0.088 |  |
| >3 | 3 | 35.2 | Fixed | -0.29 (-0.57, 0.00) | **0.047** |  |
| Rehabilitation type |  |  |  |  |  | 0.452 |
| Multicomponent | 2 | 0 | Fixed | -0.37 (-0.67, -0.06) | **0.018** |  |
| Exercise only | 4 | 7.1 | Fixed | -0.21 (-0.50, 0.09) | 0.174 |  |
| **Barthel's ADL** | | | | SMD |  |  |
| Intervention initiation time after surgery (months) | | | |  |  | - |
| ≤3 | 3 | 58.7 | Random | 0.22 (-0.34, 0.78) | 0.445 |  |
| >3 | 1 | - | - | - | - |  |
| Intervention duration (months) | | | |  |  | - |
| ≤3 | 4 | 0 | Fixed | -0.10 (-0.31, 0.12) | 0.369 |  |
| >3 | 1 | - | - | - | - |  |
| Rehabilitation type |  |  |  |  |  | 0.178 |
| Multicomponent | 2 | 79.1 | Random | 0.30 (-0.34, 0.94) | 0.361 |  |
| Exercise only | 3 | 0 | Fixed | -0.18 (-0.48, 0.11) | 0.216 |  |
| **Instrumental ADL** | | | | SMD |  |  |
| Intervention initiation time after surgery (months) | | | |  |  | - |
| ≤3 | 4 | 0 | Fixed | 0.21 (-0.05, 0.46) | 0.114 |  |
| >3 | 1 | - | - | - | - |  |
| Intervention duration (months) | | | |  |  | 0.806 |
| ≤3 | 3 | 0 | Fixed | 0.13 (-0.17, 0.42) | 0.407 |  |
| >3 | 2 | 0 | Fixed | 0.18 (-0.12, 0.48) | 0.244 |  |
| Rehabilitation type |  |  |  |  |  | 0.806 |
| Multicomponent | 3 | 0 | Fixed | 0.18 (-0.11, 0.70) | 0.244 |  |
| Exercise only | 3 | 0 | Fixed | 0.13 (-0.17, 0.42) | 0.407 |  |
| **SPPB** |  |  |  | SMD |  |  |
| Intervention initiation time after surgery (months) | | | |  |  | 0.096 |
| ≤3 | 3 | 83.9 | Random | 0.52 (-0.06, 1.09) | 0.077 |  |
| >3 | 2 | 0 | Fixed | -0.02 (-0.27, 0.24) | 0.897 |  |
| Intervention duration (months) | | | |  |  | - |
| ≤3 | 1 | - | - | - | - |  |
| >3 | 4 | 84.6 | Random | 0.38 (-0.10, 0.85) | 0.125 |  |
| Rehabilitation type |  |  |  |  |  | 0.793 |
| Multicomponent | 3 | 89.4 | Random | 0.35 (-0.38, 1.08) | 0.342 |  |
| Exercise only | 2 | 71 | Random | 0.24 (-0.20, 0.68) | 0.287 |  |
| **Fast gait speed** | | | | SMD |  |  |
| Intervention initiation time after surgery (months) | | | |  |  | - |
| ≤3 | 1 | - | - | - | - |  |
| >3 | 3 | 48.2 | Fixed | 0.20 (-0.09, 0.50) | 0.179 |  |
| Intervention duration (months) | | | |  |  | 0.266 |
| ≤3 | 3 | 77.4 | Random | 0.57 (-0.22, 1.37) | 0.158 |  |
| >3 | 2 | 0 | Fixed | 0.09 (-0.23, 0.40) | 0.583 |  |
| Rehabilitation type |  |  |  |  |  | **0.018** |
| Multicomponent | 2 | 0 | Fixed | 0.02 (-0.23, 0.26) | 0.903 |  |
| Exercise only | 3 | 41.6 | Fixed | 0.63 (0.18, 1.07) | **0.006** |  |
| **Usual gait speed** | | | | SMD |  |  |
| Intervention initiation time after surgery (months) | | | |  |  | 0.365 |
| ≤3 | 2 | 0 | Fixed | 0.00 (-0.21, 0.22) | 0.977 |  |
| >3 | 7 | 0 | Fixed | 0.14 (-0.07, 0.35) | 0.181 |  |
| Intervention duration (months) | | | |  |  | 0.491 |
| ≤3 | 7 | 0 | Fixed | 0.13 (-0.08, 0.33) | 0.330 |  |
| >3 | 3 | 0 | Fixed | 0.03 (-0.15, 0.21) | 0.755 |  |
| Rehabilitation type |  |  |  |  |  | 0.436 |
| Multicomponent | 4 | 0 | Fixed | 0.04 (-0.12, 0.20) | 0.649 |  |
| Exercise only | 6 | 0 | Fixed | 0.16 (-0.10, 0.41) | 0.226 |  |
| **6MWT** |  |  |  | SMD |  |  |
| Intervention initiation time after surgery (months) | | | |  |  | - |
| ≤3 | 0 | - | - | - | - |  |
| >3 | 4 | 52.3 | Random | 0.37 (-0.19, 0.92) | 0.199 |  |
| Intervention duration (months) | | | |  |  | - |
| ≤3 | 3 | 0 | Fixed | 0.67 (0.12, 1.23) | **0.017** |  |
| >3 | 1 | - | - | - | - |  |
| Rehabilitation type |  |  |  |  |  | - |
| Multicomponent | 1 | - | - | - | - |  |
| Exercise only | 3 | 0 | Fixed | 0.67 (0.12, 1.23) | **0.017** |  |
| **Falls Efficacy Scale** | | | | SMD |  |  |
| Intervention initiation time after surgery (months) | | | |  |  | - |
| ≤3 | 5 | 78.8 | Random | 0.39 (-0.08 0.86) | 0.104 |  |
| >3 | 1 | - | - | - | - |  |
| Intervention duration (months) | | | |  |  | 0.794 |
| ≤3 | 4 | 84.7 | Random | 0.27 (-0.39, 0.92) | 0.429 |  |
| >3 | 2 | 61.2 | Random | 0.37 (-0.08, 0.83) | 0.109 |  |
| Rehabilitation type |  |  |  |  |  | **0.002** |
| Multicomponent | 2 | 51.9 | Random | 0.91 (0.43, 1.39) | **<0.001** |  |
| Exercise only | 4 | 0 | Fixed | 0.07 (-0.13, 0.28) | 0.486 |  |
| **Knee extensor strength** | | | | SMD |  |  |
| Intervention initiation time after surgery (months) | | | |  |  | 0.133 |
| ≤3 | 6 | 30.3 | Fixed | 0.18 (0.03, 0.34) | **0.019** |  |
| >3 | 3 | 0 | Fixed | 0.47 (0.13, 0.82) | **0.007** |  |
| Intervention duration (months) | | | |  |  | **0.028** |
| ≤3 | 3 | 0 | Fixed | 0.69 (0.26, 1.11) | **0.002** |  |
| >3 | 6 | 0 | Fixed | 0.18 (0.03. 0.33) | **0.019** |  |
| Rehabilitation type |  |  |  |  |  | **0.015** |
| Multicomponent | 2 | 0 | Fixed | 0.03 (-0.19, 0.24) | 0.801 |  |
| Exercise only | 7 | 0 | Fixed | 0.38 (0.20, 0.56) | **<0.001** |  |
| **SF-36 MCS** |  |  |  | SMD |  |  |
| Intervention initiation time after surgery (months) | | | |  |  | - |
| ≤3 | 3 | 0 | Fixed | 0.08 (-0.15, 0.31) | 0.486 |  |
| >3 | 0 | - | - | - | - |  |
| Intervention duration (months) | | | |  |  | - |
| ≤3 | 0 | - | - | - | - |  |
| >3 | 3 | 0 | Fixed | 0.08 (-0.15, 0.31) | 0.486 |  |
| Rehabilitation type |  |  |  |  |  | - |
| Multicomponent | 3 | 0 | Fixed | 0.08 (-0.15, 0.31) | 0.486 |  |
| Exercise only | 0 | - | - | - | - |  |
| **SF-36 PCS** |  |  |  | SMD |  |  |
| Intervention initiation time after surgery (months) | | | |  |  | 0.470 |
| ≤3 | 3 | 0 | Fixed | 0.46 (0.23, 0.69) | **<0.001** |  |
| >3 | 3 | 0 | Fixed | 0.68 (0.13, 1.23) | **0.016** |  |
| Intervention duration (months) | | | |  |  | 0.470 |
| ≤3 | 3 | 0 | Fixed | 0.68 (0.13, 1.23) | **0.016** |  |
| >3 | 3 | 0 | Fixed | 0.46 (0.23, 0.69) | **<0.001** |  |
| Rehabilitation type |  |  |  |  |  | 0.470 |
| Multicomponent | 3 | 0 | Fixed | 0.46 (0.23, 0.69) | **<0.001** |  |
| Exercise only | 3 | 0 | Fixed | 0.68 (0.13, 1.23) | **0.016** |  |
| **Emergency department visit** | | | | RR |  |  |
| Intervention initiation time after surgery (months) | | | |  |  |  |
| ≤3 | 4 | 52.2 | Random | 0.85 (0.51, 1.42) | 0.533 |  |
| >3 | 0 | - | - | - | - |  |
| Intervention duration (months) | | | |  |  |  |
| ≤3 | 1 | - | - | - | - |  |
| >3 | 3 | 52.1 | Random | 0.78 (0.48, 1.25) | 0.297 |  |
| Rehabilitation type |  |  |  |  |  | **0.019** |
| Multicomponent | 2 | 0 | Fixed | 0.62 (0.43, 0.90) | **0.012** |  |
| Exercise only | 2 | 0 | Fixed | 1.52 (0.90, 2.89) | 0.206 |  |
| **Falls** |  |  |  | RR |  |  |
| Intervention initiation time after surgery (months) | | | |  |  | - |
| ≤3 | 6 | 9.6 | Fixed | 0.93 (0.76, 1.14) | 0.478 |  |
| >3 | 1 | - | - | - | - |  |
| Intervention duration (months) | | | |  |  | 0.344 |
| ≤3 | 3 | 0 | Fixed | 0.18 (0.83, 1.40) | 0.574 |  |
| >3 | 5 | 22.1 | Fixed | 0.91 (0.73, 1.14) | 0.422 |  |
| Rehabilitation type |  |  |  |  |  | 0.766 |
| Multicomponent | 6 | 16.2 | Fixed | 0.96 (0.80, 1.16) | 0.699 |  |
| Exercise only | 2 | 0 | Fixed | 1.03 (0.70, 1.52) | 0.885 |  |
| **Hospital readmission** | | | | RR |  |  |
| Intervention initiation time after surgery (months) | | | |  |  | - |
| ≤3 | 5 | 0 | Fixed | 0.75 (0.57, 0.98) | **0.035** |  |
| >3 | 0 | - | - | - | - |  |
| Intervention duration (months) | | | |  |  | 0.897 |
| ≤3 | 2 | 58.8 | Random | 0.83 (0.27, 2.58) | 0.745 |  |
| >3 | 4 | 0 | Fixed | 0.77 (0.59, 1.02) | 0.069 |  |
| Rehabilitation type |  |  |  |  |  | 0.523 |
| Multicomponent | 4 | 24.3 | Fixed | 0.92 (0.71, 1.18) | 0.492 |  |
| Exercise only | 2 | 14.3 | Fixed | 0.77 (0.48, 1.24) | 0.275 |  |
| **Walking outdoors** | | | | RR |  |  |
| Intervention initiation time after surgery (months) | | | |  |  | - |
| ≤3 | 1 | - | - | - | - |  |
| >3 | 2 | 0 | Fixed | 0.83 (0.50, 1.38) | 0.468 |  |
| Intervention duration (months) | | | |  |  | - |
| ≤3 | 3 | 28.4 | Fixed | 1.00 (0.83, 1.21) | 0.984 |  |
| >3 | 1 | - | - | - | - |  |
| Rehabilitation type |  |  |  |  |  | - |
| Multicomponent | 3 | 0 | Fixed | 1.06 (0.87, 1.28) | 0.573 |  |
| Exercise only | 1 | - | - | - | - |  |

^#^ Subgroup comparison.

ADL: activities of daily living; PCS: physical component score; RR: risk ratio; SF-36: Short Form-36 questionnaire; SMD: standardized mean difference; SPPB: short physical performance battery;
