# Supplementary material for: Effectiveness of home-based exercise for functional rehabilitation in older adults after hip fracture surgery: A systematic review and meta-analysis of randomized controlled trials
Source: PLoS One. 2024 Dec 19;19(12):e0315707. doi: 10.1371/journal.pone.0315707 (PMC11658508; doi:10.1371/journal.pone.0315707)
Supplement: S8 Table — (DOCX) [file pone.0315707.s009.docx]

S8 Table. Egger’s test for publication bias.

| Outcomes | Z | P |
| --- | --- | --- |
| Berg balance score | 0.93 | 0.353 |
| Timed-up-and-go test | 0.84 | 0.401 |
| Barthel's ADL | -0.10 | 0.924 |
| Instrumental ADL | 0.56 | 0.578 |
| SPPB | 0.32 | 0.751 |
| Fast gait speed | 2.72 | **0.007** |
| Usual gait speed | 1.26 | 0.209 |
| Falls efficacy scale | -0.44 | 0.663 |
| Knee extensor strength | 2.66 | **0.008** |
| SF-36 PCS | 0.60 | 0.545 |
| Falls | 1.01 | 0.315 |
| Hospital readmission | -0.94 | 0.348 |

ADL: activities of daily living; PCS: physical component score; SF-36: Short Form-36 questionnaire; SPPB: short physical performance battery.
