# Supplementary figures and images for: Effectiveness of home-based exercise for functional rehabilitation in older adults after hip fracture surgery: A systematic review and meta-analysis of randomized controlled trials
Source: PLoS One. 2024 Dec 19;19(12):e0315707. doi: 10.1371/journal.pone.0315707 (PMC11658508; doi:10.1371/journal.pone.0315707)

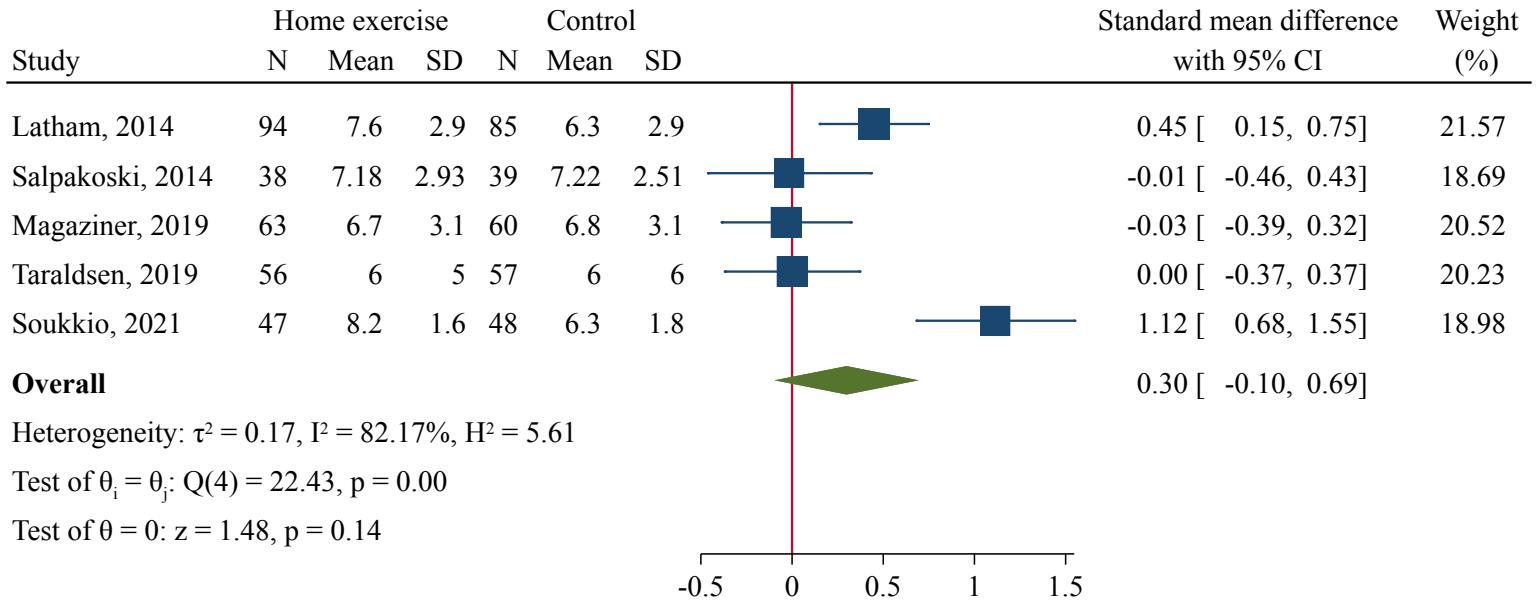

Random-effects DerSimonian-Laird model

Supplement: S1 Fig — (PDF) [file pone.0315707.s010.pdf]

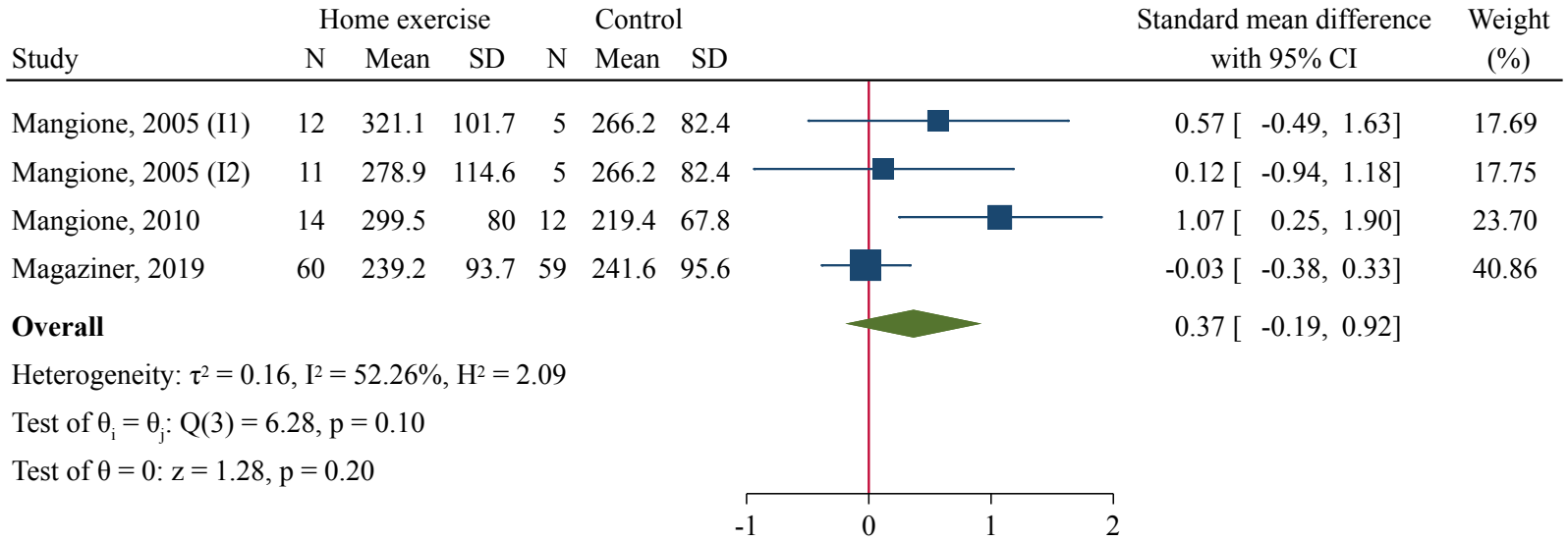

Random-effects DerSimonian-Laird model

Supplement: S2 Fig — (PDF) [file pone.0315707.s011.pdf]

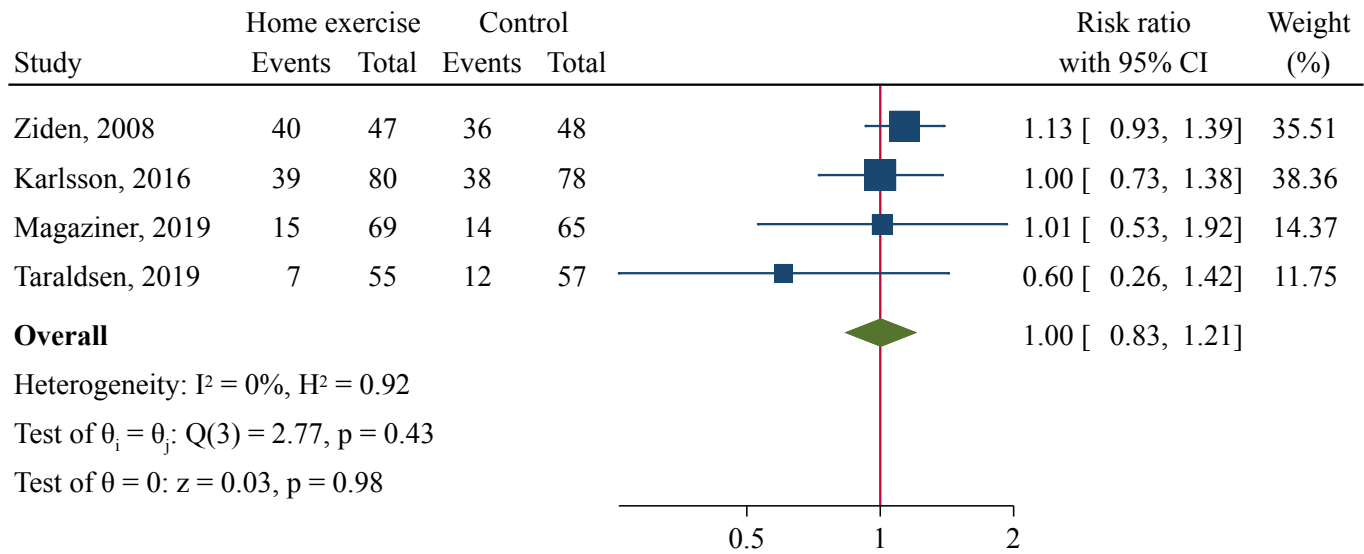

Fixed-effects Mantel-Haenszel model

Supplement: S3 Fig — (PDF) [file pone.0315707.s012.pdf]

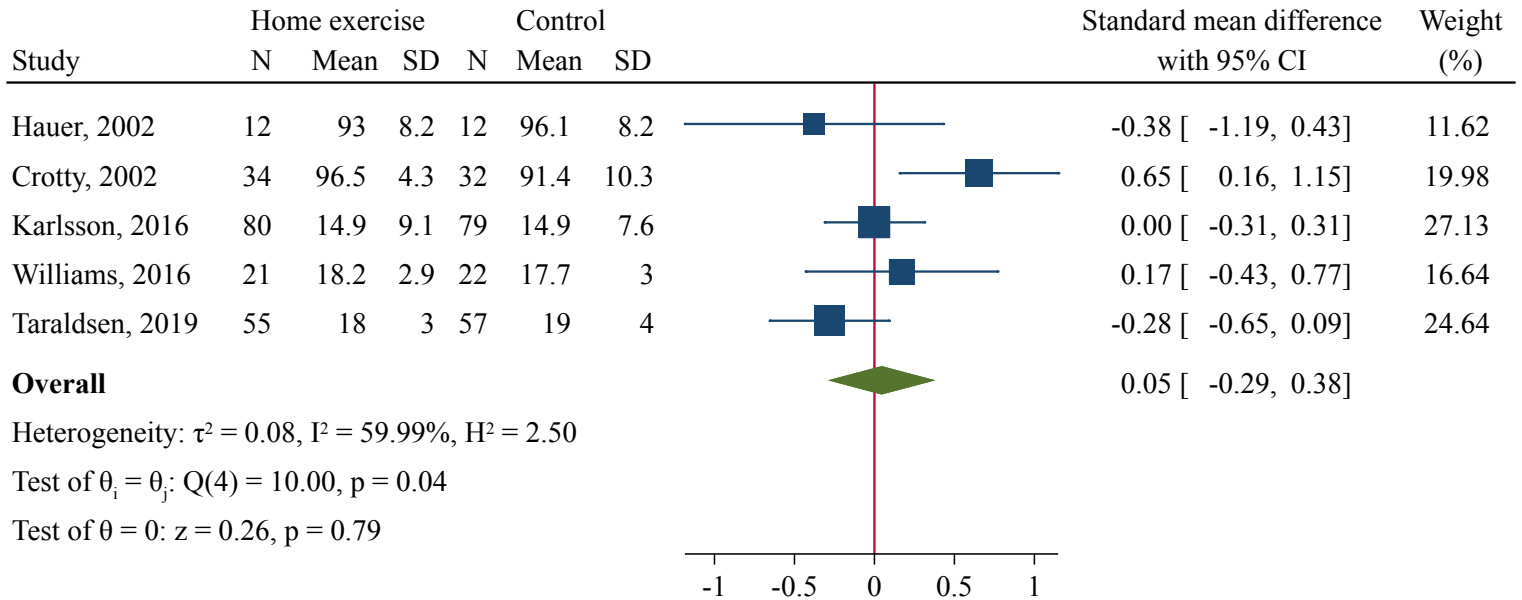

Random-effects DerSimonian-Laird model

Supplement: S4 Fig — (PDF) [file pone.0315707.s013.pdf]

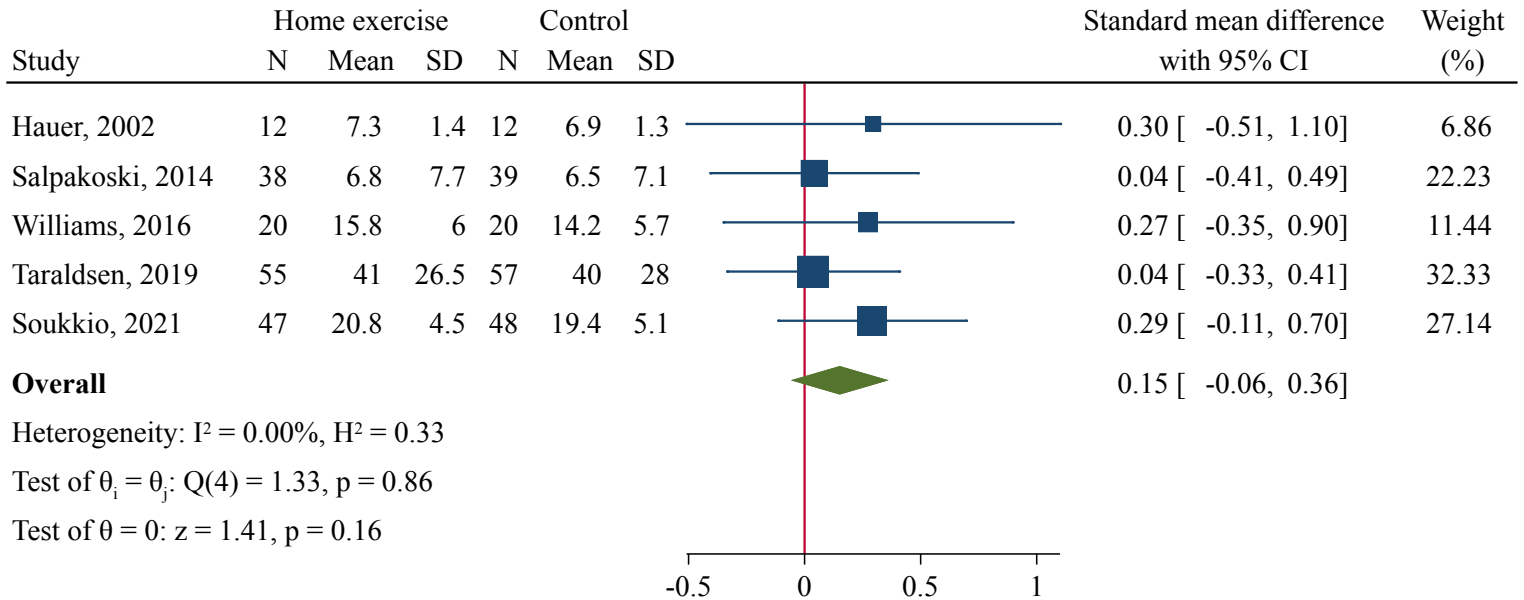

Fixed-effects inverse-variance model

Supplement: S5 Fig — (PDF) [file pone.0315707.s014.pdf]

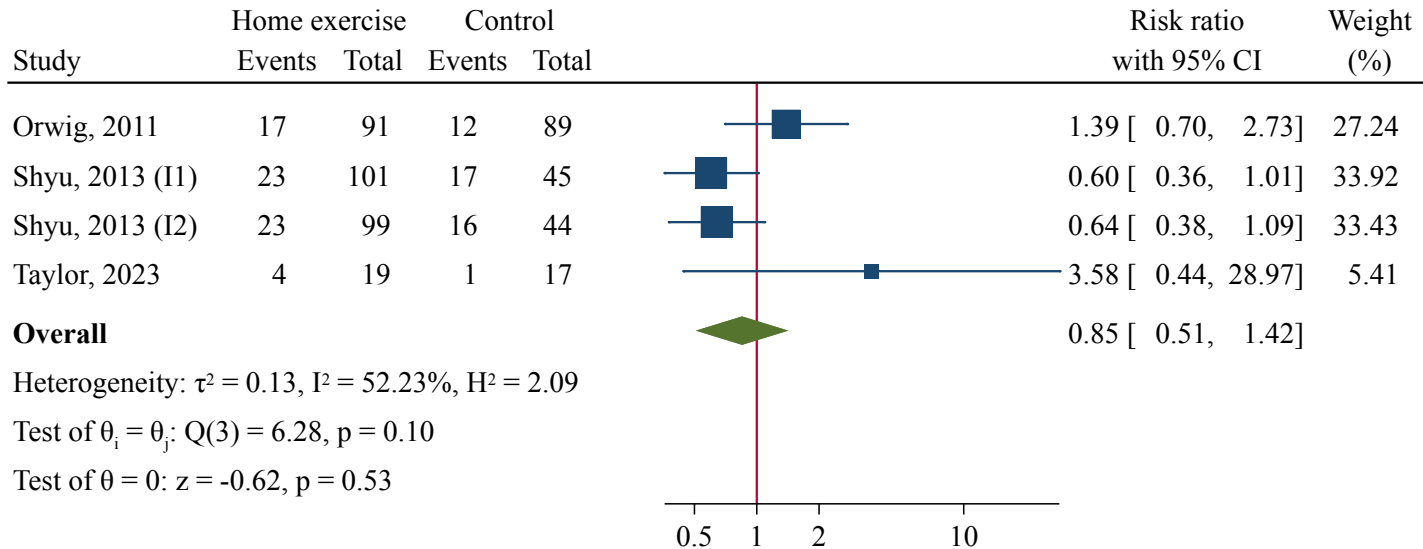

Random-effects DerSimonian-Laird model

Supplement: S6 Fig — (PDF) [file pone.0315707.s015.pdf]

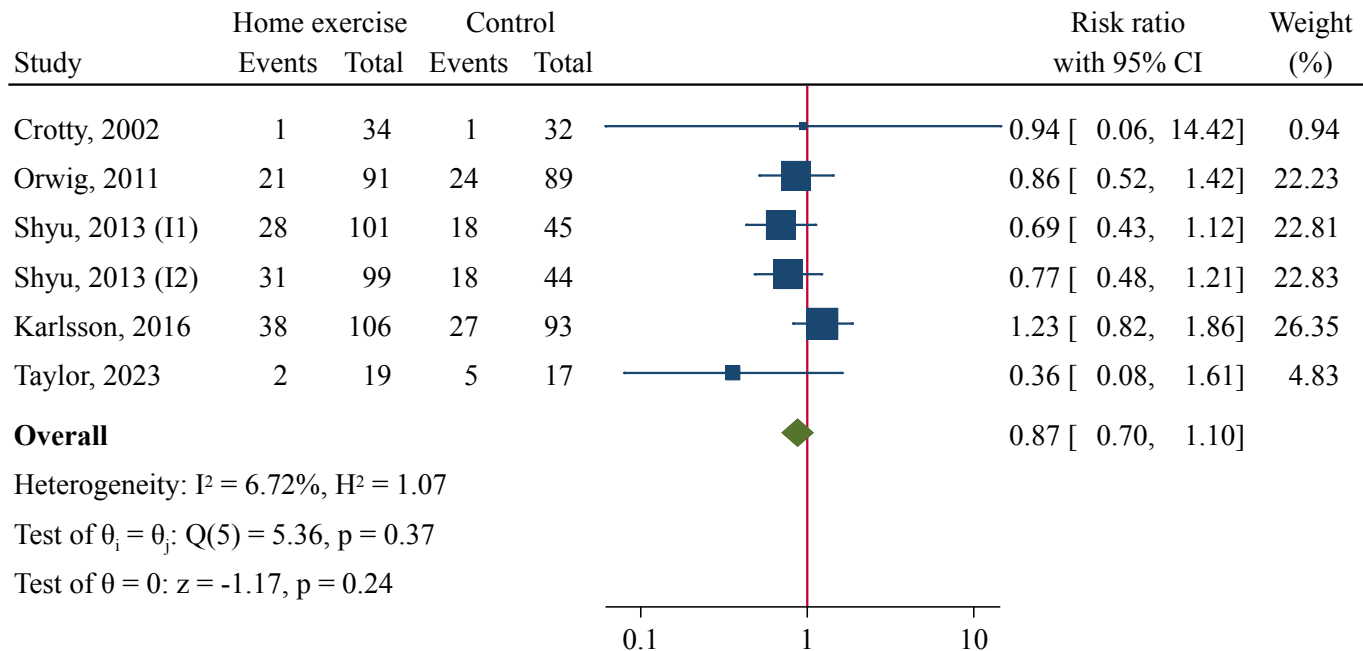

Fixed-effects Mantel-Haenszel model

Supplement: S7 Fig — (PDF) [file pone.0315707.s016.pdf]

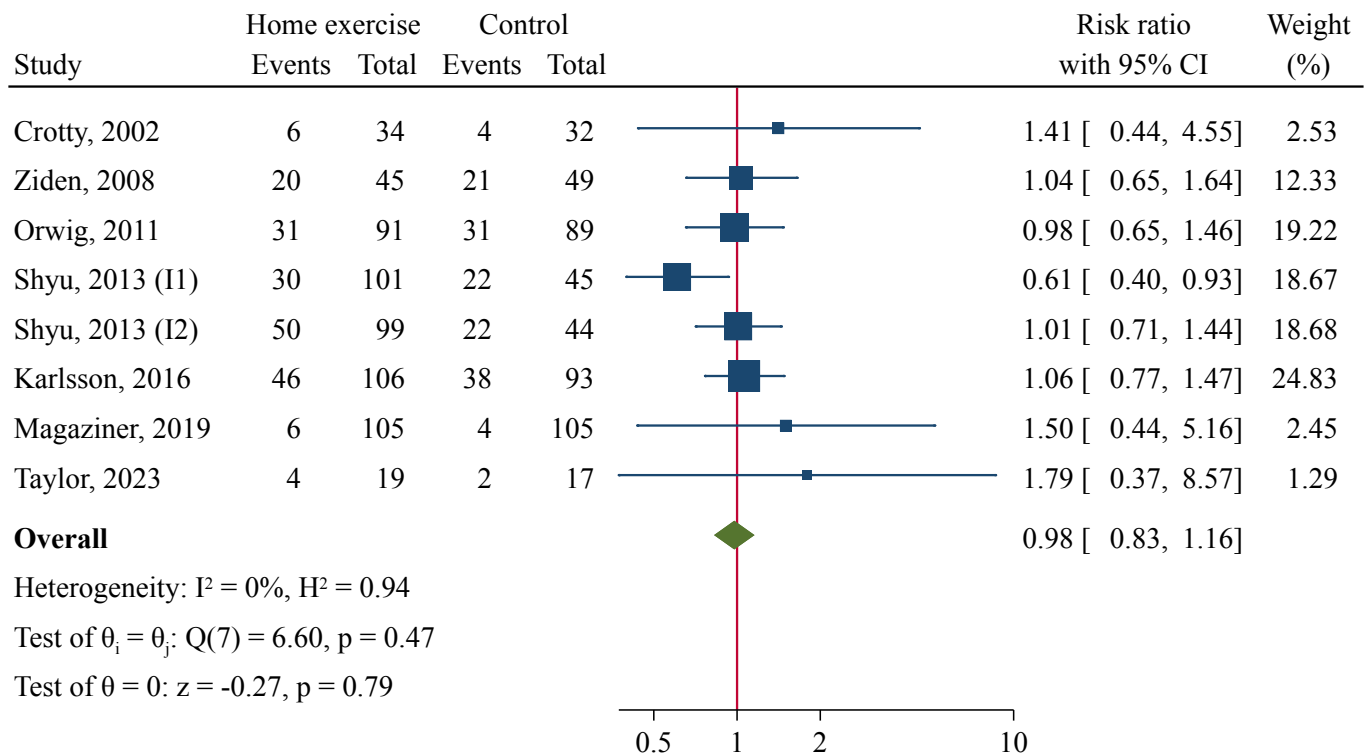

Fixed-effects Mantel-Haenszel model

Supplement: S8 Fig — (PDF) [file pone.0315707.s017.pdf]

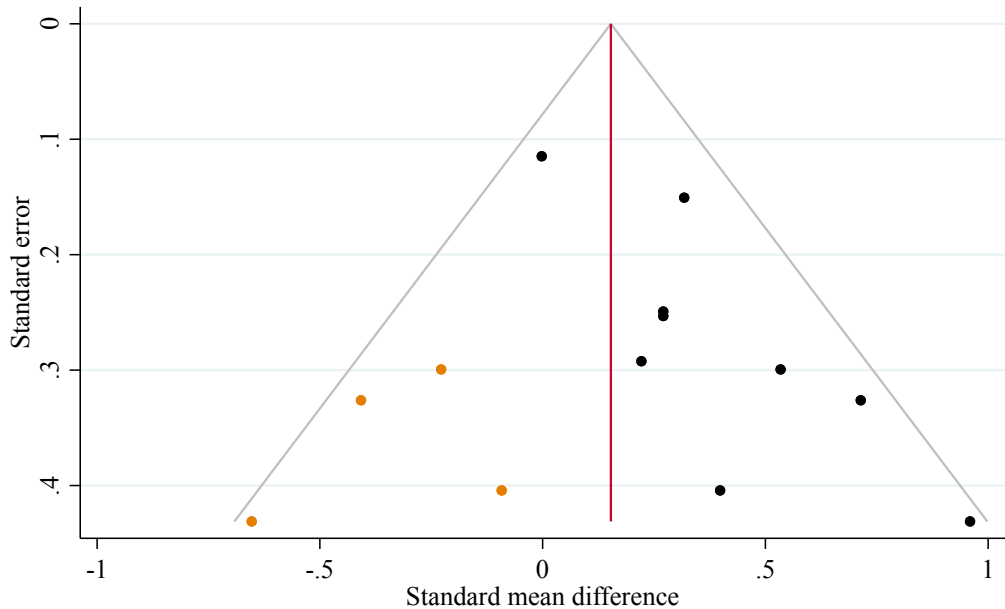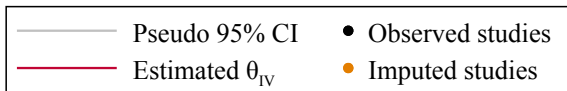

Supplement: S9 Fig — (PDF) [file pone.0315707.s018.pdf]
